# GBS‐MeDIP: A method for the simultaneous identification of genetic and epigenetic variants in tumors

**DOI:** 10.1002/2211-5463.70360

**Published:** 2026-11-09

**Authors:** Fábio Pértille, Shiva Rezaei, Julia Uffenorde, Oliver Gimm, Mohammad Ali Hosseinpour Feizi, Luiz Lehmann Coutinho, Carlos Guerrero‐Bosagna

**Affiliations:** ^1^ Physiogy and Environmental Toxicology Program Institute of Organismal Biology, Uppsala University Sweden; ^2^ Avian Behavioral Genomics and Physiology Group, Department of Physics, Chemistry and Biology (IFM) Linköping University Sweden; ^3^ Animal Biotechnology Laboratory, Animal Science Department University of São Paulo (USP)/Luiz de Queiroz College of Agriculture (ESALQ) Brazil; ^4^ Faculty of Natural Sciences, Department of Animal Biology University of Tabriz Iran; ^5^ Department of Medical Biochemistry and Cell Biology, Sahlgrenska Center for Cancer Research, Institute of Biomedicine, Sahlgrenska Academy University of Gothenburg Sweden; ^6^ Department of Surgical Science Uppsala University Sweden; ^7^ Division of Surgery, Orthopedics and Oncology (KOO), Department of Clinical and Experimental Medicine (IKE) Linköping University Hospital Sweden

**Keywords:** epigenetics, GBS‐MeDIP, genetics, genomic and epigenomic profiling, marker

## Abstract

Tumor development arises from combined genetic and epigenetic alterations, yet most sequencing approaches examine these layers independently. We present GBS‐MeDIP, a low‐input and cost‐efficient method that combines genotype‐by‐sequencing with methylated DNA immunoprecipitation to jointly identify single nucleotide polymorphisms (SNPs), copy number variations (CNVs), and differentially methylated regions (DMRs) within the same genomic fraction. The method was evaluated in two clinical contexts: gastric adenocarcinoma (a malignant epithelial tumor) and parathyroid adenoma (a benign endocrine tumor). In both case studies, we detected thousands of SNPs, with principal component analysis (PCA) revealing genetic divergence in gastric tumors and substantial inter‐individual genetic background differences in parathyroid samples. We identified many CNVs in both datasets, with CNVs more frequent in parathyroid tumors, showing a stronger representation in promoter regions than SNPs. Gastric cancer showed a balanced methylation profile, with four DMRs reaching false discovery rate (FDR) significance, whereas Mimpara‐treated parathyroid tumors displayed widespread hypermethylation, with all significant DMRs hypermethylated. These significant DMRs mapped to genes involved in immune regulation, chromatin function, DNA repair, and endocrine signaling. Annotation analysis demonstrated that, in both tissues, CNVs were enriched in promoter and exonic regions, whereas DMRs were preferentially located in intronic and intergenic regions, reflecting the CpG‐enriched reduced genomic fraction accessed by GBS‐MeDIP. Importantly, both datasets contained DMRs overlapping CNVs, highlighting the method's ability to reveal genetic confounders in methylation studies. Together, these results show that GBS‐MeDIP enables combined genomic and epigenomic profiling in heterogeneous clinical tumor samples.

## Graphical abstract

GBS‐MeDIP combines DNA sequencing with enrichment of methylated DNA to profile genetic and epigenetic variation in the same reduced genomic fraction. Applied to gastric cancer and parathyroid tumors, it identifies sequence variants, copy number changes, and differentially methylated regions together, helping distinguish true epigenetic changes from effects related to underlying genetic variation.

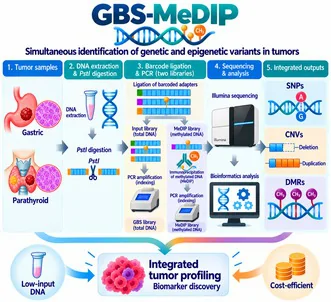

**Abbreviations**
CNV: Copy number variation
DMR: Differentially methylated region
FDR: False discovery rate
GCT: Gastric cancer tissue
GMT: Gastric marginal tissue
GO: Gene Ontology
GWAS: Genome‐wide association study
MeDIP: Methylated DNA immunoprecipitation
NGS: Next‐generation sequencing
PC: Parathyroid tissue from placebo‐treated control patients
PCA: Principal component analysis
PT: Parathyroid tissue from Mimpara‐treated patients
RAD‐seq: Restriction site‐associated DNA sequencing
RE: Restriction enzyme
ROI: Region of interest
RRBS: Reduced‐representation bisulfite sequencing
RRL: Reduced‐representation library
SNP: Single nucleotide polymorphism
TSS: Transcription start site
UTR: Untranslated region

## Background

Epigenetic modifications involve chemical interactions of molecules with DNA nucleotides (e.g., cytosines) and/or proteins that are involved in its compaction (i.e., histones) [1]. These interactions influence gene expression, allowing the genome to be differentially active depending on developmental stage, tissue type, or disease influence [2]. DNA methylation involves the addition of methyl groups to DNA bases, mainly cytosines neighboring guanines, dinucleotides known as CpG sites [3]. This interaction may remain stable throughout multiple cell divisions [1]. DNA methylation, as an essential epigenetic modification, can influence gene expression [4]. DNA hypo‐methylation generally leads to gene activation, chromosomal instability, upregulation of proto‐oncogenes, increasing of mutation rates and recombination, suppression of X‐chromosome inactivation, and suppression of genomic imprinting [5]. On the other hand, DNA hyper‐methylation is often associated with genomic instability (through silencing of the DNA repairing genes) and gene silencing resulting in down‐regulation of tumor suppressor genes and chromatin condensation [6]. It also plays a key role in the suppression of transposable element activity to maintain genome integrity [7].

Tumors, both malignant and benign, are heterogeneous and develop from the combination of genetic mutations and epigenetic abnormalities [8]. The identification of diagnostic and prognostic molecular biomarkers aiming for an early and accurate detection represents promising approaches to improve the treatment and survival of cancer patients [9, 10]. Combined genomic and epigenomic profiling can be of major help in such identification. Combinations of various kinds of biomarkers including genetic, epigenetic, and cancer‐specific antigens are shown to positively influence the efficacy of tumor diagnosis [11]. DNA methylation‐based signatures are valuable epigenetic detection biomarkers because of their potential for early detection of tumorigenesis, identification of malignancy types, as well as for tracking tumor recurrence after surgery [12, 13]. Recent research has uncovered hypermethylated variants that can be used as potential epigenetic markers for cancer detection. Examples include *MGMT* as a potential therapy choice marker in glioma [14], *MLH1* as a forecaster marker for cisplatin therapy outcome in colon cancer [11], *GSTP1* as a diagnostic element in prostate cancer [15], and a number of others [11].

Research is rapidly advancing to find specific and sensitive cancer detection panels based on DNA methylation. Recently, hypermethylation of the promoter region of the *SEPT9* gene has passed the process of Food and Drug Administration (FDA) certificate approval to be considered as a clinical test for screening of colon cancer [16]. Another methylation‐based test is Bladder EpiCheck, distributed by Nucleix, which evaluates more than 15 methylation markers to detect bladder cancer recurrence with a specificity of 88% [17, 18]. Oncgnostics GmbH™ (Jena, DE) has developed “GynTect®,” an epigenetic biomarker test applied in cervical smears to screen for cervical cancer [18]. IvyGene™™ (West Lafayette, IN) is a blood‐based cancer test that allows detecting hypermethylation of target sites in cell‐free DNA across various cancers such as breast, colon, liver, and lung tumors [18]. Other DNA methylation‐based detection biomarkers have already received CE‐IVD, FDA PMA approval [18].

Besides epigenetic alterations, genetic variants such as the commonly investigated single nucleotide polymorphisms (SNPs) and also copy number variations (CNVs) may indicate susceptibility to cancer progression and even change the efficiency of drugs for cancer treatments [19, 20, 21, 22]. For example, mutations in the *MLH1* and *MSH2* genes are risk factors for colon cancer development [23] and many genetic mutations are currently used in cancer screening such as in *BRCA1* and *BRCA2* genes in relation to the detection of breast, ovarian, and prostate cancers [24, 25]. Additionally, CNV studies have reported amplified variants in oncogenes like *MITF* in the case of melanoma malignancy, and *NKX2‐1* in lung cancers [25]. CNVs are recently reported to be the most important genetic alteration occurring across different cancer types [22].

High‐throughput DNA genetic and epigenetic profiling can help to uncover genome‐wide variants in SNPs, CNVs, and differentially methylated regions (DMRs) to pave the way for the identification of markers of tumor development, patient prognosis, or individual response to specific treatments. Because the cost of whole‐genome interrogation across many individuals is still a limiting factor when aimed at being used in large numbers of individuals, reduced representation libraries become a good alternative [26, 27]. The need for high‐throughput and genome‐wide parallel interrogation of genetic and methylomic variants in cancer research (and many other biological fields) led us to develop a method [28] that combines two previously described techniques, namely Genotype‐by‐Sequencing (GBS) [29] and Methylated DNA Immunoprecipitation (MeDIP) [30, 31]. GBS is a genotyping approach that uses Restriction Enzyme (RE) to reduce the genome complexity followed by the addition of barcodes to individual samples. The use of RE allows access to regions of the genome that are not easily reached by sequence capture methods [32]. This happens because lower copy number regions (common in species with high genetic variations [32]) are enriched with much higher efficiency when using a restriction enzyme (RE) compared with random shearing. GBS was initially described in maize but has currently been applied in many different animal models, both inbred and outbred [26, 27, 29, 33, 34, 35, 36]. In addition, GBS is used as a standard method for genetic map construction in non‐model species [36, 37, 38]. MeDIP, in turn, is a genome‐wide method in which an anti‐5‐methylcytosine antibody is used to capture methylated DNA sheared fragments [39]. MeDIP is one of the most widely used methods to evaluate genome‐wide DNA methylation and has excellent performance for genome coverage in comparison with equivalent methods [40].

The combination of these techniques, called GBS‐MeDIP [28], has been developed as a cost‐efficient protocol for parallel genotyping and epigenotyping of equivalent reduced genomic fractions across large numbers of individual DNA samples. Although the full protocol for the GBS‐MeDIP has been published elsewhere [28], the current paper describes its optimization, limitations, and its applications for tumor research. The GBS‐MeDIP takes advantage of RE fragmentation and barcoding of individual DNA samples performed in the GBS, which allows pooling them for the later bioinformatic identification of individual DNA fragments [32, 41]. Then, this pool of barcoded fragmented samples is subjected to MeDIP [28], followed by PCR amplification, purification of PCR products, and sequencing on the Illumina platform. In addition to substantial decreases in sequencing costs, this method increases the accuracy of statistical analyses by increasing the statistical power as a consequence of the large number of samples that can be analyzed [29].

The present paper shows the application of the GBS‐MeDIP in two case studies to identify methylomic and genetic variations (SNPs and CNVs) in two types of human tumors among sample groups. These groups include (a) human gastric cancerous tissue samples (GCT) and their matched marginal tissue samples, which were used as controls (GMT), and (b) human parathyroid tissues from patients medicated with the drug “Mimpara” (PT) and untreated patients, used as controls (PC). Mimpara works as a calcimimetic and is responsible for activating calcium‐sensing receptors in order to inhibit secretion of the parathyroid hormone [42].

## Methods

### Sample collection and DNA extraction

For Case Study I, Gastric adenocarcinoma tissues and the corresponding non‐cancerous marginal tissues were collected from patients during endoscopy procedure in Tabriz hospitals, (East Azerbaijan province, Tabriz, Iran). All the tissues were immediately frozen in liquid nitrogen and stored at −80 °C for future experiments. Written consent was obtained from all participants before the collection of samples. Patients confirmed their voluntary participation in the study, and written informed consent was obtained from all participants before sample collection. The study was approved by the Ethics Committee of Tabriz University of Medical Sciences (approval no. IR.TBZMED.REC.1397.772), and all procedures were conducted in accordance with the principles of the Declaration of Helsinki. We selected patients without any type of prior radiotherapy or chemotherapy treatments. DNA extraction was performed using Qiagen DNeasy blood & tissue kit (cat. no.: 69504) according to the manufacturer's protocol. The quality and quantity of the extracted DNAs, A260/230 and A260/280 ratios, were assessed using a NanoDrop 2000c spectrophotometer (Thermo Fisher Scientific, Waltham, MA, USA).

For Case Study II, parathyroid tissues were collected from patients following parathyroidectomy operated on for primary hyperparathyroidism at Linköping University hospital. All the tissues were immediately frozen in liquid nitrogen and stored at −80 °C for future experiments. Patients confirmed their voluntary participation in the study. Written consent was obtained from all participants before the collection of samples. Since these samples were collected at Linköping University Hospital, the approvals were almost certainly issued by the Regional Ethical Review Board in Linköping (Regionala etikprövningsnämnden i Linköping), which later became the Swedish Ethical Review Authority. The patients were randomly selected from a larger group of patients operated on for primary hyperparathyroidism ensuring that both patients with and without prior Mimpara treatment were included. DNA extraction was performed using Qiagen DNeasy blood & tissue kit (kat. no.: 69504) according to manufacturer's protocol. The quality and quantity of the extracted DNAs, A260/230 and A260/280 ratios, were confirmed by NanoDrop 2000c spectrophotometer (Thermo Fisher Scientific, Waltham, MA, USA).

### GBS‐MeDIP

The main steps for GBS‐MeDIP library preparation are: (i) DNA digestion, (ii) adaptor and barcode ligation, (iii) PCR of the GBS fraction for the creation of the genomic library and quality control, (iv) MeDIP on the remaining pooled sample, followed by PCR, (v) purification of both the genomic and methylomic libraries, (vi) sequencing, and (vii) bioinformatic processing and analysis. The detailed protocol has been described in Rezaei et al. [28].

### DNA denaturation and digestion

Different RE were evaluated *in silico*, including PstI, MseI, ApeKI, SbfI, MspI, and EcoRI. However, PstI yielded the best results and was used for the case studies in this paper. In order to improve the performance of the RE in the human genome, a DNA denaturation step of 95 °C for 10 min was added, followed by immersion in ice for 5 min before applying RE digestion. In human samples, this was a crucial step for successful digestion. This denaturation step was introduced to minimize potential interference from DNA‐associated proteins with *PstI* activity. Next, *PstI* (10 U/μL; Thermo Fisher Scientific, cat. no. ER0611) was used to digest DNA samples. with 16 h of incubation (overnight). Although the output of the digestion reaction requires 100 ng of DNA, 400 ng DNA was used as initial material, sufficient for three distinct GBS MeDIP experiments. The digestion products were divided into three aliquots, providing material for replicate experiments or additional optimization if required. For the initial implementation of the protocol, one aliquot can be used to test the workflow while the remaining aliquots are retained as backup material.

In order to check the quality of the digestion process as well as a suitable size fragmentation for Illumina sequencing (200–500 bp), *PstI* digestion products were confirmed with gel electrophoresis by running 2 μL of each digested DNA sample on 1.5% agarose gels. In case of incomplete digestion, it is essential to repeat the digestion process.

### Adaptor ligation to digested DNAs

After the DNA is digested by the enzyme, two Illumina adapters are added at the ends of each DNA fragment produced [28]. This is a crucial step for the clustering of Illumina probes within the sequencing flowcell. One of the adapters is called the “*common adapter*,” which is the same for all the samples, and the other is called the “*barcode adapte*r,” which allows for the later identification of individual DNA sequences across different samples through bioinformatic analyses [41]. A script designed by Thoma P. van Gurp (https://github.com/thomasvangurp/GBS_barcodes) was used for controlling the nucleotide balance when designing the barcodes.

For the ligation reaction, adaptors containing defined barcodes are used to attach to the cohesive ends produced by the *PstI* cleavage. The barcode adapters end with a 5 to 9 bp barcode on the 5′ end of the minus strand and a 4 bp overhang on the 3′ end of the plus strand, which is complementary to the sticky ends created after *PstI* digestion. The nucleotide sequences of these Illumina adapters are: 5′ACACTCTTTCCCTACACGACGCTCTTCCGATCTNNNNNTGCA3′ and 3′TGTGAGAAAGGGATGTGCTGCGAGAAGGCTAGANNNNN5′, where NNNNN denote the barcode and barcode complement sequences. The common adapter has a *PstI*‐complementary sticky end on the 3′ end of the minus strand. The nucleotide sequences are as follows: 3′ACGTTCTAGCCTTCTCGCCAAGTCGTCCTTACGGCTC5′ and 5′AGATCGGAAGAGCGGTTCAGCAGGAATGCCGAG3′.

After the ligation of the adapters, the samples are pooled, and the methylated fraction of the whole pool is subsequently captured by MeDIP. This captured genomic fraction enriched for DNA methylation is then PCR amplified. However, only DNA fragments containing both adapters are able to be amplified and subsequently sequenced [41].

### Checking the efficiency of DNA/barcodes ligation by GBS‐PCR

To examine the efficacy of adaptor ligation, a quality validation of the ligation reaction needs to be performed via PCR using specific primers targeting both Illumina adaptors. This means that the PCR reaction will amplify only DNA fragments ligated to the Illumina adapters in the previous step. Thus, if the binding step is successful, the ligated and amplified fragments can then be detected in an agarose gel after electrophoresis. The GBS PCR product is then purified with magnetic beads before being sent for Next Generation Sequencing (NGS).

### MeDIP

The MeDIP procedure was performed according to a recent optimization of the method [31]. The required reagents for the MeDIP have been previously described [31] but are also described later in our method paper [28].

### GBS‐MeDIP PCR reaction

In order to perform the PCR reaction, a suitable number of PCR cycles was first identified with the aim of obtaining a minimal amount of dimeric sub‐products. For this, real‐time quantitative PCR amplification curves were monitored using Applied Biosystems 7500 Real‐Time PCR System (Thermo Fisher Scientific, Waltham, MA, USA), and the end of the early non‐exponential phase was considered of interest, as it represents the maximum amplification obtained where the PCR product is still on a linear increase in relation to the input. Based on this, 23 cycles were used as the suitable number of PCR cycles for performing the amplification of the libraries produced with the GBS‐MeDIP method on the human DNA samples.

PCR amplification was performed using DreamTaq DNA polymerase (Thermo Fisher Scientific, USA, cat. no. EP0703). The preparation of the PCR reaction mix is performed according to the manufacturer's protocol. The temperature, number, and length of cycles were adjusted in order to avoid primer dimers and optimize the amplification of the desired fragments.

### Purification before sequencing

The PCR purification is an essential step that needs to be carefully performed to achieve a pure library, minimizing the presence of primer‐dimers and other small fragments. A high‐efficiency purification approach based on solid‐phase reversible immobilization magnetic beads from the AMPure XP kit (Beckman Coulter, cat. no. A63881) is employed to clean the DNA libraries produced in both the GBS and GBS‐MeDIP PCRs [43].

### Quality and quantity assessment of the GBS‐MeDIP library

In this step, library fragment sizes were assessed using an Agilent 2100 Bioanalyzer (Agilent Technologies, Santa Clara, CA, USA) with the Agilent High Sensitivity DNA Kit (cat. no. 5067‐4626). One microliter of the final purified GBS MeDIP‐PCR product is sufficient to examine whether the final amplification of the libraries is successful.

### Next generation sequencing

As previously stated, a barcoding system is used in the GBS method involving a barcode adapter (in order to identify each one of the individual samples) and a common adapter suitable for Illumina sequencing. These attach to the ends of the digested DNA fragments [41]. Because of this, the methylation and genomic signals associated with each individual sample can be distinguished after the sequencing process. The successfully libraries obtained after amplification and purification are paired‐end sequenced (125 cycles) on an Illumina HiSeq 2500 sequencing system (Illumina, San Diego, CA, USA) using the HiSeq SBS v4 Kit. Sequencing was performed at the SNP&SEQ facilities of SciLifeLab (Uppsala, Sweden).

### Bioinformatic processing and analysis

The CASAVA (Illumina) program was used for the initial processing of the samples by converting the “.bcl” (base call files) to the “.fastq” extension that are compatible with the programs used for the read's alignment. Stored FASTQ format files related to each read contain an identification key, a barcode encompassing the relevant information of the respective sample. The individual barcodes and the debris of the *PstI* cut site containing the sequence 5′ CTGCA 3′, help in distinguishing a desired read. From the .fastq file, the expected fragments are categorized into individual files, which correspond to individuals identified by their respective barcodes. The quality of the reads was checked using FastQC v.0.11.3 [44]. Quality trimming was performed in short read sequences during the data process. Both for SNP/CNV calling and for methylation analyses, quality‐trimmed reads were aligned against the human reference sequence (GRCh38/hg38, UCSC Genome Browser) by selecting the “very sensitive‐local alignment” option in the Bowtie2 tool v.2.2.5 pipeline [45] using default parameters for paired‐end reads. *PstI*‐reduced genome is used for both SNP calls and as input. The input works as genomic background correction in epigenetic analysis. Samtools v.0.1.19 [46] was used to evaluate the coverage depth of each sample. Following read alignment, the data were analyzed through two complementary workflows according to the biological signal being investigated. The GBS libraries were used for SNP and CNV detection, whereas the GBS‐MeDIP libraries were used for DNA methylation analysis, using the corresponding GBS libraries as genomic input for background correction. While CNV and DNA methylation analyses share the same preprocessing workflow and rely on comparisons of regional read counts between experimental groups, SNP identification is performed independently for each individual by genotype calling based on allele frequencies. Consequently, these molecular features require different downstream analytical and normalization procedures and cannot be processed using identical statistical criteria.

SNP calling was performed using Tassel v.3.0 program [47] following the default TASSEL‐GBS Discovery Pipeline, with the following filtering parameters: 1% for minimum minor allele frequency (mnMAF), 70% of minimum taxon call rate (mnTCov) and 70% of site call rate (mnScov) for SNP calling using the reduced genome data. This procedure for the SNP call has been previously described in chicken [29]. To identify SNPs associated with the experimental groups, two complementary association tests were applied: the Cochran–Armitage trend test (TREND), which evaluates associations between genotype dosage and group membership, and the allelic association test (ALLELIC), which compares allele frequencies between groups. Both tests were used to identify genetic variants associated with the respective tissue comparisons. SNPs with *P* ≤ 0.05 were considered nominally significant for downstream analyses.

Regarding the CNV and methylated DNA sequencing data, the Stacks v.1.39 [48] program was used to perform data de‐multiplexing and quality trimming of reads using default parameters. DMR call is done by using reduced and methylated genome while *PstI*‐reduced genome is used as input for genomic background correction and for CNV call. For CNV calling, the aligned sequence files (bam) of each individual (from each treatment) are merged into unique files. The “view” option from Samtools v.1.3.14 [46] is used to generate a “hit” file from each unique file containing the coverage information for each base pair sequenced from each treatment. This “hit” file is then used for CNV calling by the CNV‐Seq tool [46] across the human genome using default parameters. For DMR call, after data alignment, the Macs2 (v.2.1.1) [49] tool was used. Macs2 is a recommended tool to identify sample‐wise ‘peak specific’ methylated regions of variable sizes in experiments using paired control to determine enrichment against background [49, 50, 51]. This program was used to combine the sequences and define the peaks (FDR ≤ 0.05) of sequencing coverage between the cases and their controls, as previously described [52]. Then, a file containing the positions of each peak, called “regions of interest” (ROI), is used as input in downstream analyzes. The BSgenome.Hsapiens.UCSC.hg38 package was employed as the reference genome from the Bioconductor R repository. For basic data processing, quality controls, normalization as well as the recognition of differential methylation regions (DMRs), the MEDIPS R‐package v1.50.0 and edgeR v3.40.2 were used. The MEDIPS package provides a maximum number of stacked reads per genomic position resulting in avoiding the possible artifacts caused by PCR amplification. This is achieved by the Poisson genome‐wide distribution of the stacked reads. A threshold of *P* = 0.001 was used as the criterion for the detection of stacked reads. The advantage of the ROI BED file obtained from Macs2 peak calling over the MEDIPS is the capability of ROI to allow large methylated regions that are not divided into adjacent smaller pre‐defined windows (usually 300 bps length size). Therefore, the analysis is “peak specific” and more straightforward. ROI identification and differential group testing were performed as two sequential statistical steps. First, methylated regions meeting the ROI‐level FDR threshold of ≤ 0.05 were retained. Differential methylation between experimental groups was subsequently tested within these retained ROIs using edgeR as implemented in MEDIPS. A nominal *P*‐value ≤ 0.05 was used to identify DMRs, and FDR ≤ 0.05 indicated those remaining significant after correction for multiple testing. The conversion of the MeDIP data into genome‐wide relative methylation scores is proceeded through a CpG dependent normalization [53]. The dependence between short read coverage and CpG density at windows throughout the genome is the basis for the normalization process [54]. This relationship can be visualized as a calibration plot. A calibration plot was obtained by using one of the individuals of each test to generate a coupling set (object that groups information about CpG density genome‐wide) to each specific treated vs. control test. According to this scale, a threshold for a minimum sum of counts across all samples per window is determined (minRowSum = 10 is the default). For both CNVs and DMRs, gain and loss categories were defined to represent the direction of corresponding log2 fold changes (logFC) between experimental groups. Positive values indicate relative increases in copy number (CNVs) and methylation gains (hypermethylated DMRs) in GCT compared to GMT in the gastric dataset, and in PT compared to PC in the parathyroid dataset, while negative values represent decreased copy numbers (CNVs) and methylation losses (hypomethylated DMRs).

For all sets of coordinates (SNPs, DMRs, and CNVs), genomic context was assigned with the ChIPseeker v1.34.1 R package using the UCSC knownGene transcript set for hg38 (TxDb.Hsapiens.UCSC.hg38.knownGene) and org.Hs.eg.db.The annotatePeak function was run with a promoter definition of Transcription Start Site (TSS) ±3000 bp (tssRegion = c (−3000, 3000)), obtaining feature labels (promoter, untranslated region [UTR], exon, intron, downstream, distal intergenic) and distances to the nearest TSS on GRCh38/hg38.

#### Per‐chromosome densities and correlation

For each tissue (gastric and parathyroid) and feature type (DMR, CNV), genomic intervals stored as GRanges were tallied per chromosome using the table(seqnames()) function. Counts were normalized by chromosome size obtained from the seqlengths() function to yield densities (features per megabase). To evaluate whether methylation and copy number variation covary across chromosomes, Pearson's correlation (cor.test) was computed between DMR_perMb and CNV_perMb within each tissue.

#### Genomic annotation proportions

The detailed ChIPseeker labels (e.g., “Promoter (<=1 kb),” “Intron,” “Distal Intergenic,” “5′UTR/3′UTR,” “Exon”) were collapsed by string matching into six classes: Promoter, Exon, UTR, Intron, Intergenic, and Other. For each tissue × feature type, the number of loci (N) and the proportion per class (Percent = 100 N/total) were computed; the results were combined across datasets.

#### CpG content and CpG density

For each interval, the CpG count recorded in the CG metadata column and the interval length (width) were used to calculate (i) mean CpG per region and (ii) CpG density (CpG/kb = CG ÷ [width/1000]). Means were summarized by the six annotation classes separately per tissue for DMRs and CNVs.

#### Side‐by‐side comparison of DMRs vs CNVs by annotation

To facilitate direct comparison within each tissue, a table joining DMR and CNV counts and percentages by annotation class was constructed to report the differences in percentage (ΔPercent = CNV%−DMR%).

#### Functional convergence across genomic and epigenomic features

To investigate convergence among the genetic and epigenetic features identified by GBS‐MeDIP, SNPs with *P* ≤ 0.05 from the TREND or ALLELIC association tests were retained, with duplicated genomic loci removed, while DMRs with edgeR *P* < 0.05 and the final called CNV regions were used for downstream comparisons. Physical overlaps among SNPs, CNVs, and DMRs was assessed separately for the gastric and parathyroid datasets using the findOverlaps function from the GenomicRanges R package. In parallel, genes associated with each feature type were compared to identify shared genes associate to SNPs and CNVs, SNPs and DMRs, CNVs and DMRs, and across all three feature types. Gene Ontology (GO) Biological Process enrichment analysis was subsequently performed on each shared gene set using the enrichGO function from the clusterProfiler R package, with a False discovery rate (FDR) ≤ 0.05 considered significant. In addition, overlapping genes in only two of the feature classes (SNP, CNV or DMR) were combined into a multi‐feature‐supported gene set and subjected to the same GO Biological Process enrichment analysis separately. To reduce redundancy among significant GO terms, semantic similarity analysis was performed using the rrvgo R package with the Rel similarity method and org.Hs.eg.db annotation database. GO terms with a semantic similarity ≥0.7 were grouped, using −log10(FDR) as the ranking score, and representative biological processes were selected from the resulting semantic clusters. Semantic similarity networks were generated from significant GO terms, with nodes representing enriched biological processes and edges connecting terms with Rel semantic similarity ≥ 0.7. Node size was scaled according to −log10(FDR), semantic clusters were distinguished by color, and the representative term selected for each cluster was highlighted. Cross‐feature and functional convergence analyses were performed in R v4.2.0 using Bioconductor v3.16, GenomicRanges v1.50.2, clusterProfiler v4.6.2, rrvgo v1.10.0, and org.Hs.eg.db v3.12.0. Semantic similarity networks were constructed using igraph v2.3.2 and ggraph v2.1.0.

## Results

### GBS‐MeDIP enzyme selection

Among the different REs evaluated *in silico*, including *PstI*, *MseI*, *ApeKI, SbfI, MspI*, and *EcoRI*, *PstI* was the most efficient in enriching for genomic regions of a suitable length size for sequencing (ranging from 200 to 500 bps). *PstI* recognizes the 5′CTGCAG 3′ motif, generating fragments with overhangs and cutting the genome in a methylation insensitive manner, as this motif does not contain CpG sites [29]. In chickens, a broad distribution of the *PstI* generated fragments was observed across the genome [29]. To identify the suitability of using *PstI* with the genomes of other vertebrates, *in silico* digestions (using the *PstI* restriction site) of the genome of *Danio rerio, Gallus, Mus musculus, Homo sapiens*, *Sus scrofa*, and *Canis familiaris* were performed (Fig. S1a–f). The selection of these species was based on the animal models we investigated [29, 35, 52, 55, 56, 57, 58, 59] and species widely used in genomic studies [60, 61]. The fragment pattern obtained by these *in silico* digestions indicated that *PstI* cleavage of each of these genomes produces high quantities of fragments of the desired length (ranging 200–500 bp) for Illumina sequencing platforms (Fig. S1).

After the *in silico* digestions of the same six model species genomes, fragments with at least one CpG in their composition were identified. This analysis simulates what would happen if an entire genome were methylated to the fullest, subjected to MeDIP, and subsequently cleaved by *PstI*. With the human genome, the *PstI in silico* digestion generated 178 002 fragments ranging 200–500 bp, which corresponds to 14% of all the fragments obtained. More than 90% of the fragments ranging 200–500 bp have at least one CpG dinucleotide in their composition (Fig. 1G). This shows an enrichment of CpG fragments after *PstI* cleavage as recently reported [52]. In comparison, *PstI* digestion of the chicken genome generated 171 959 fragments with at least one CpG, which corresponds to ~ 20% of all the fragments generated. Importantly, fragments smaller than 200 bp produced by the *PstI* cleavage are less prone to being captured by the MeDIP (Fig. S1). Thus, the MeDIP step selects fragments larger than 200 bp, which are also well suited for Illumina sequencing.

**Fig. 1:**
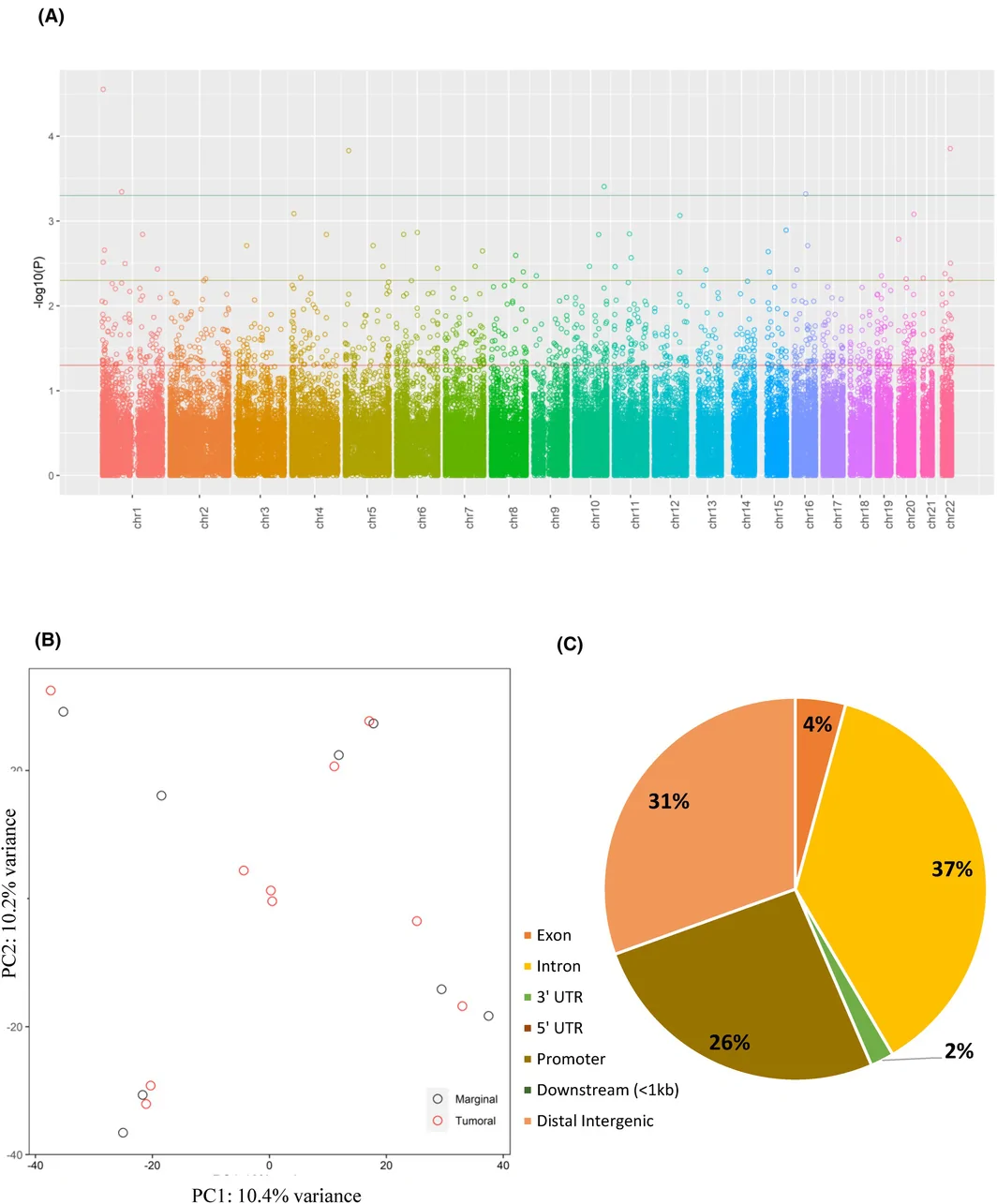
Plots related to the single nucleotide polymorphisms (SNPs) identified between gastric cancer tissue (GCT) and gastric margin tissue (GMT) DNA samples from 18 individuals. (A) Manhattan plot showing the SNPs identified with different levels of significance (green line indicates a *P*‐value of 0.05 for nominal significance, yellow line for a *P*‐value of 0.005 indicating higher significance, and red line for a *P*‐value of 0.1, suggesting an exploratory threshold); (B) principal component analysis (PCA) based on all the 48 894 SNPs identified; and (C) pie chart showing the functional annotation of the SNPs identified (*P* ≤ 0.05).

### Demonstration of GBS‐MeDIP in case studies involving human tumors

To characterize the genome‐wide distribution and features of the identified regions, chromosomal densities, annotation proportions, and CpG composition of both ROI (as putative DMRs) and putative CNVs were summarized for both the gastric tissue and parathyroid tumor datasets. Here, putative refers to all regions that could be identified using our GBS‐MeDIP approach, without applying statistical significance thresholds.

Across the 24 chromosomes examined, DMR and CNV densities were not significantly correlated in the gastric dataset (Pearson's *r* = 0.209, *P* = 0.327), whereas a moderate positive correlation was observed in the parathyroid dataset (r = 0.630, P < 0.001; Data S1).

Both DMRs and CNVs were preferentially recovered from CpG‐rich genomic contexts. CNVs were concentrated within promoter and exonic regions, which together represented roughly 45%–50% of all putative CNV regions, reflecting the high CpG representation of the fragments generated by this restriction enzyme. In contrast, ROI were more broadly distributed, with approximately 65% located in intronic and intergenic regions, consistent with the larger genomic space of these features and the stochastic methylation variation typically found outside promoter islands.

A strong negative correlation (*r* ≈ −0.80) was observed between mean CpG count and CpG density (CpG/kb) across annotations, both in the gastric (*r* = −0.80) and parathyroid (*r* = −0.83) datasets. This correlation was calculated using annotation‐specific averages (promoter, exon, intron, intergenic, UTR, and other categories) from the DMR and CNV regions identified in each dataset. This arises because CNV regions, which are much larger than DMRs, contain many CpGs spanning wide low‐CpG segments with reduced CpG density, while DMRs, particularly those near promoters, harbor fewer CpGs overall but at much higher local density. This size‐dependent effect indicates that absolute CpG counts reflect the total CpG content of a region, whereas CpG/kb reflects how densely CpGs are distributed along it (see Data S1).

In both the gastric and parathyroid datasets, promoters and exons showed the highest CpG densities (CpG/kb), whereas introns and intergenic regions displayed much lower values. This pattern accounts for the stronger promoter enrichment of CNVs and the broader genomic distribution of DMRs. Detailed annotation percentages are provided in Data S1 (side‐by‐side DMR vs. CNV summary).

Next, the GBS‐MeDIP is employed in two types of human tumor samples to investigate genetic and epigenetic changes, presented here in the following case studies:

### Case study I

Gastric cancer is a high mortality malignancy that has been ranked as the fourth most common cancer in the world [62]. Gastric cancer is a multifactorial disease that results from the interplay between genetic and epigenetic factors [63]. Worldwide variation in gastric cancer incidence can be due to environmental factors, genetic composition, and/or the human lifestyle [63]. The 5‐year survival rate of patients diagnosed with gastric cancer is currently very low, estimated to be between 20 and 30% [64]. Almost all gastric cancers involve precursor developmental epigenetic abnormalities alongside genetic changes, which eventually result in the loss of function of tumor suppressor genes and in the gain of function of oncogenes [65, 66]. DNA methylation can be aberrant (too high or too low) in cancer cells [67]. DNA methylation alterations in the promoters of tumor suppressor genes are shown to suppress their activity [68] and play a significant role in the pathogenesis of gastric cancer [69]. Additionally, studies have shown a relation between CpG island methylation and cancer progression [70]. However, DNA methylation abnormalities occurring in other genomic regions involved in regulating gene expression have not been identified. One of the advantages of the use of GBS‐MeDIP is the investigation of variants unbiased towards specific regions of the genome (e.g., promoter regions), opening the possibility for the identification of markers despite a previous description of functionality. Genome‐wide methylomic interrogation in gastric cancer tissues can help to identify biomarkers that predict cancer progression, as well as the molecular mechanism involved [71].

In this study, the genome and epigenome of surgically resected and pathologically confirmed gastric adenocarcinoma (GCT) were compared to their matched marginal (GMT) tissue [62]. Because GCTs and GMT samples were obtained as matched tissues from the same patients, this design reduced inter‐individual variation in genetic and epigenetic comparisons.

The application of our GBS‐MeDIP method led to the identification of 71 061 SNPs among all the 18 samples analyzed (10 GMT and 8 GCT; Fig. 1A). After filtering parameters were applied, 48 894 SNPs remained, which were used to perform a PCA analysis (Fig. 1B). None of these SNPs involved fixed alleles between the GCT (*N* = 8) and GMT (*N* = 10) samples. The PCA plot shows that all the samples are randomly distributed, with no distinguishable clusters of GCT or GMT samples. The PCA showed clustering of some tumoral samples in the middle, suggesting these tumors could be in a more advanced progression stage and might have acquired distinct genetic features compared to the marginal cells. The majority of the SNPs are in introns, exons and promoter regions (Fig. 1C).

Because of the importance of CNVs for the etiology of cancer, CNVs between the GCT and GMT DNA samples were investigated. A total of 27 941 CNVs (using a log2 fold change threshold of −0.6, a minimum window size of 4, and a *P*‐value threshold of 0.00001 for significance, Fig. 2A) were identified, which corresponds to 7.9% of the genome. Differences in copy number levels across tissues are shown in Fig. 2B for the CNVs with significance *P*‐value ≤ 0.00001. Modules of CNVs are clearly visible between GCT and GMT. The majority of the CNVs are in promoters, followed by intergenic, intronic, and exonic regions (Fig. 2C), a pattern that differs from that of SNPs.

**Fig. 2:**
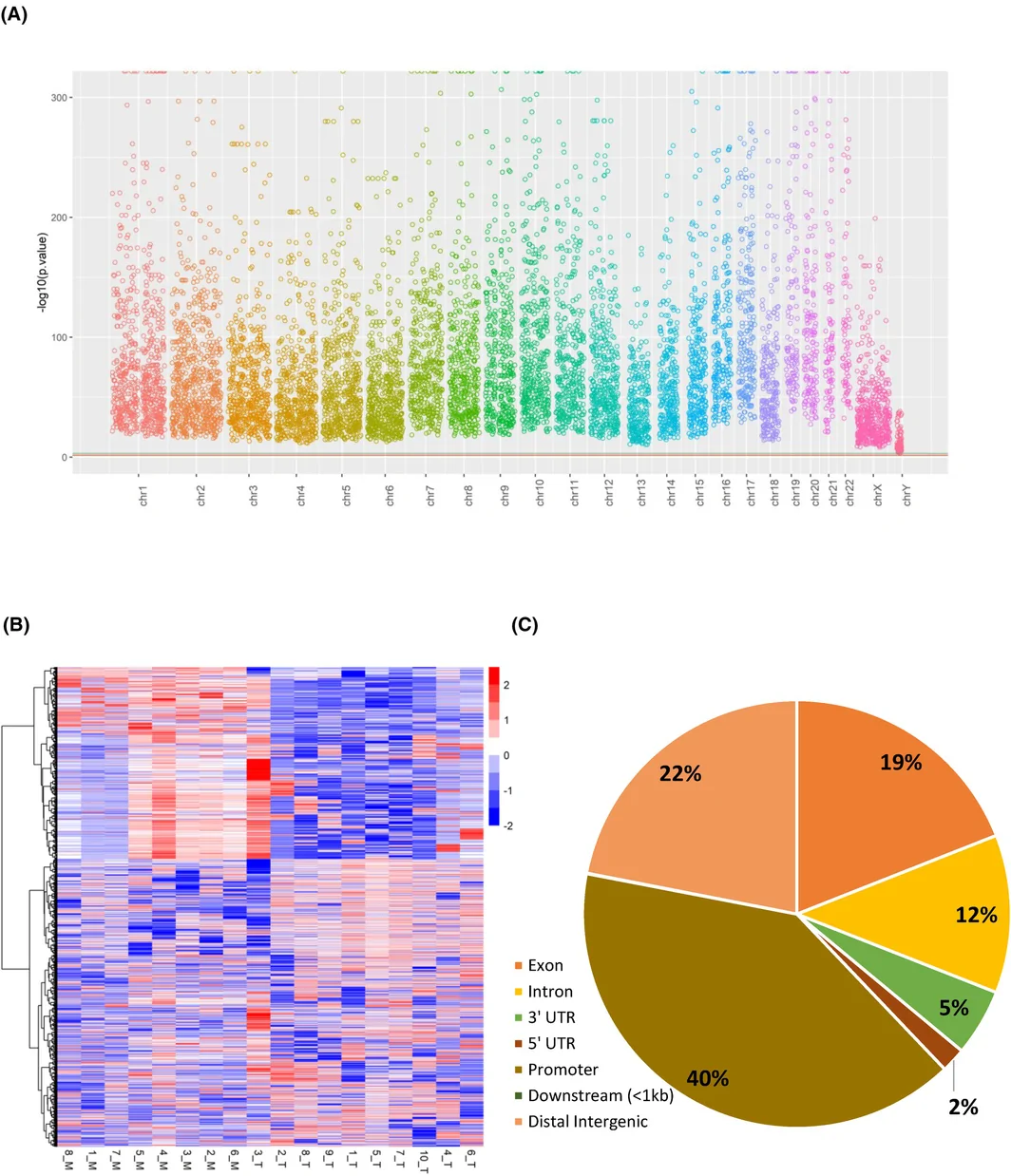
Plots related to the copy number variations (CNVs) identified between gastric cancer tissue (GCT) and gastric margin tissue (GMT) DNA samples from 18 individuals. (A) Manhattan plot showing the CNVs identified with different levels of significance; (B) Heat map showing the differences in copy number levels across individuals for CNVs obtained with *P*‐value ≤ 0.00001 (the scale to the upper right of the plot represents CNV intensity on a log2 scale, indicating relative copy number changes); and (C) pie chart showing the functional annotation of the same CNVs identified.

Next, a differential methylation analysis was performed between the GCT and GMT samples, using the GBS‐MeDIP method. A total of 2100 regions of interest were first retained employing a ROI‐level FDR threshold of ≤ 0.05. Differential comparison between GCT and GMT identified 393 DMRs at nominal *P* ≤ 0.05, of which four remained significant after FDR correction (FDR ≤ 0.05, Table 1). The DMRs related to the different levels of significance are shown in Fig. 3A. The methylation levels across tissue types are shown in Fig. 3B for the DMRs with significance *P* ≤ 0.05. There were more hypermethylated DMRs in GCT relative to GMT (Fig. 3A,B). Most of the DMRs (*P* < 0.05) are located in intronic (39%), followed by intergenic (35%), promoter (19%), exonic (5%), and 3´UTR (2%) regions (Fig. 3C). The identified DMRs were evenly distributed across the chromosomes of the human genome (Fig. 3D). In Fig. S2a, the heatmap's hierarchical clustering predominantly segregates the two groups based on significant DMRs, with the tumor cells exhibiting a more consistent methylation pattern, which further attests to the identification of methylation signatures among the majority of individuals.

**Table 1:** Genomic features and annotations for the identified DMRs (FDR≤ 0.05) between GCT and GMT DNA samples from 18 individuals. The table includes location, size, CpG count, methylation changes (logFC), statistical significance (FDR), functional context (annotation), and gene identifiers (SYMBOL and GENE NAME).

Genome location	Width	CpG count	logFC	FDR	Annotation	Symbol	Gene name
chr14:84772548–84 772 769	222	5	4.4	0.04258	Distal Intergenic	LINC02329	Long intergenic non‐protein coding RNA 2329
chr5:40886019–40 886 353	335	7	5.7	0.04243	Distal Intergenic	C7	Complement C7
chr5:71464656–71 464 788	133	4	−2.7	0.00004	Intron	BDP1	B double prime 1, subunit of RNA polymerase III transcription initiation factor IIIB
chr9:6856687–6 856 803	117	4	−3.9	0.04243	Intron	KDM4C	Lysine demethylase 4C

**Fig. 3:**
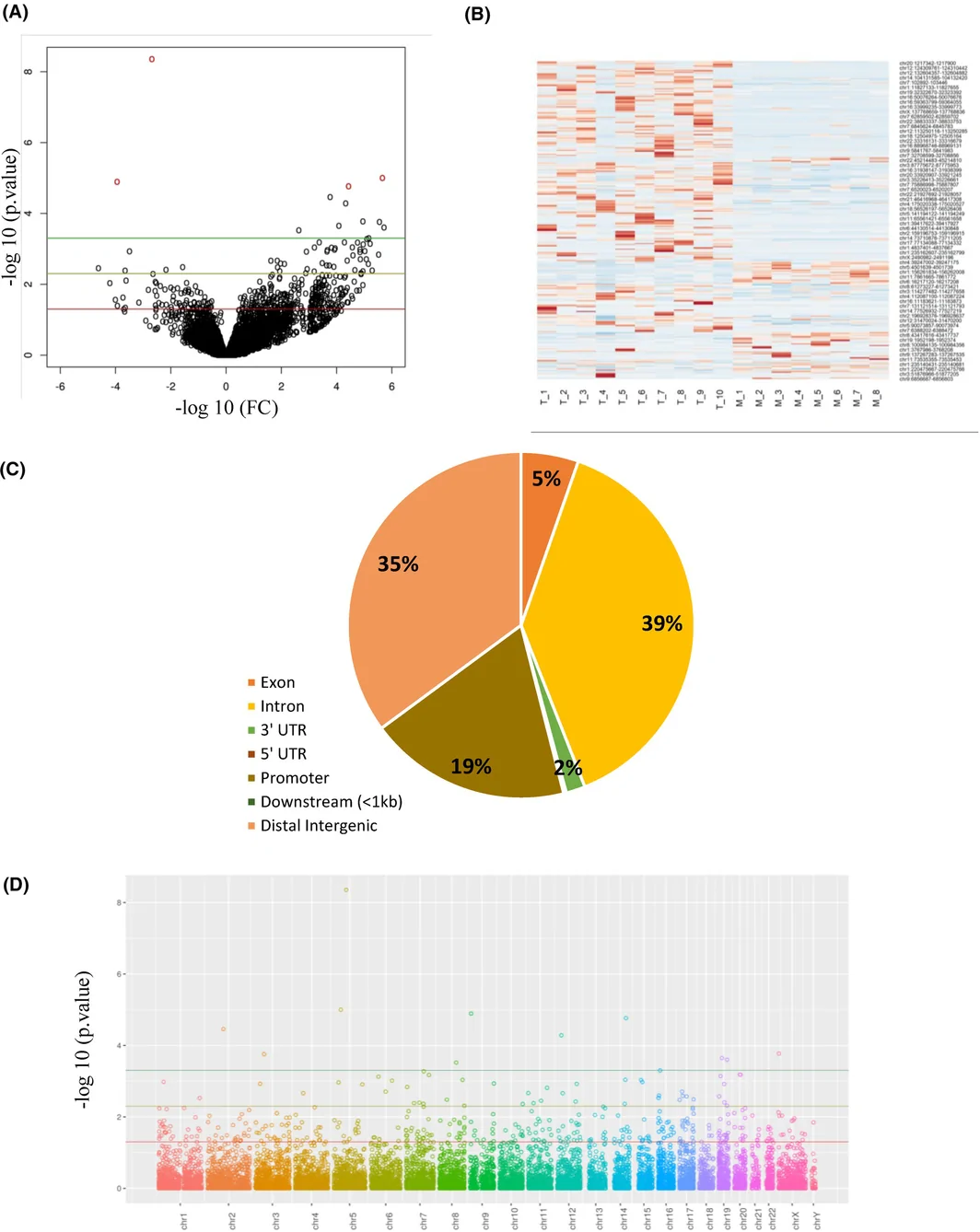
(A) Volcano plot depicting the differentially methylated regions (DMRs) identified at different levels of significance between gastric cancer tissue (GCT) and gastric margin tissue (GMT) DNA samples from 18 individuals. Positive log2 fold change indicates that tumoral cells are hypermethylated in relation to the marginal cells; thresholds correspond to *P*‐values 0.05 (red), 0.005 (yellow), and 0.0005 (green). (B) Heat map depicting methylation changes in significant DMRs (*P* ≤ 0.05); the heatmap colors show relative methylation: red for areas of hypermethylation, blue for hypomethylation, and white indicating average methylation levels across samples, reflecting the normalized baseline. (C) Pie chart representing the functional annotation of the identified DMRs (*P* ≤ 0.05). (D) Manhattan plot showing the distribution of DMRs across the chromosomes of the human genome.

To investigate the relationship among the genetic and epigenetic features identified in the gastric dataset, genomic overlaps among significant SNPs, CNVs, and DMRs were examined. Of 895 SNPs, 23 (2.57%) were located within CNV regions and one (0.11%) overlapped a DMR. Among the 393 DMRs, 10 (2.54%) overlapped CNVs (Table S1) while no SNP simultaneously overlapped both a CNV and a DMR. At the gene‐annotation level, 739, 6765, and 386 genes were associated with SNPs, CNVs, and DMRs, respectively. Among these, 243 genes were shared between SNPs and CNVs, 19 between SNPs and DMRs, and 140 between CNVs and DMRs, while eight genes were represented across all three feature types (Data S2).

Finally, GO pathway analyses were performed for the SNPs identified by the TREND and ALLELIC association tests (*P* ≤ 0.05, Fig. 4A), CNVs (*P* ≤ 0.00001, Fig. 4B) and DMRs (*P* ≤ 0.05, Fig. 4C). Gains represent increases in copy number (CNVs) and in DNA methylation (hypermethylated DMRs) in GCT compared to GMT, while losses represent reduced copy numbers and hypomethylated DMRs. These analyses revealed distinct biological pathways associated with each type of genetic or epigenetic variation. Functional convergence was also investigated by identifying overlapping genes between the CNVs, SNPs, and DMRs. GO Biological Process enrichment analysis followed by FDR correction identified significant functional enrichment among these overlapping genes comprising 70 significantly enriched GO terms (Data S2; Fig. S3). Semantic reduction of redundant GO terms grouped these into 15 representative functional clusters, including processes related to mitochondrial transport and apoptotic signaling, mRNA stability, synapse maturation, and Golgi organization and localization (Data S2; Fig. S4). However, a set of 386 genes overlapped when only two feature classes were analyzed, no GO Biological Process enrichment remained significant after FDR correction.

**Fig. 4:**
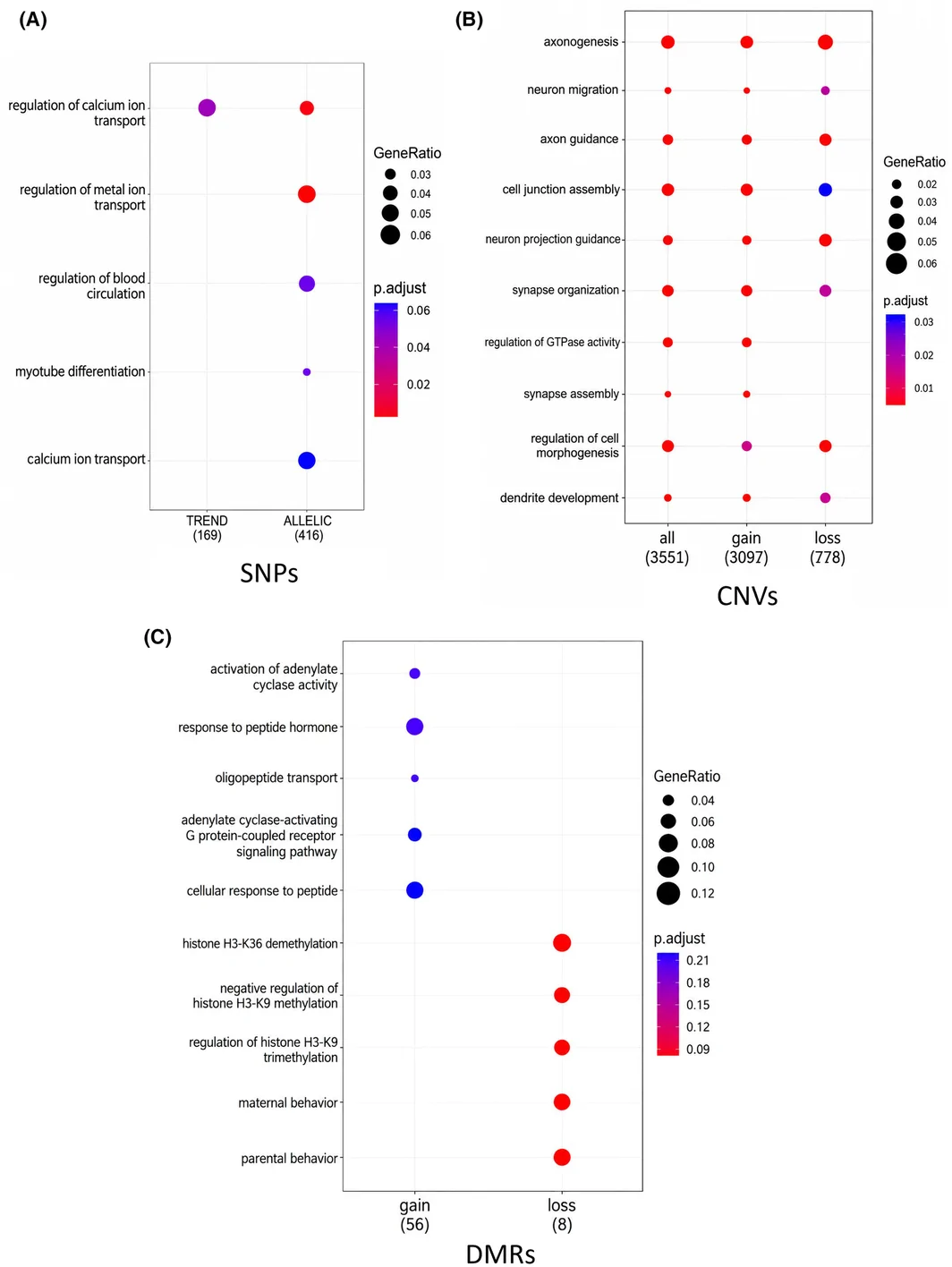
Gene Ontology (GO) pathway analyses for genomic variants in single nucleotide polymorphism (SNP), copy number variation (CNV), and differentially methylated region (DMR) categories identified between gastric cancer tissue (GCT) and gastric margin tissue (GMT) DNA samples from 18 individuals. Panel (A) shows GO terms enriched among genes associated with SNPs identified using the TREND and ALLELIC tests with a *P*‐value threshold of ≤ 0.05; panel (B) shows GO terms enriched among genes associated with CNVs (*P* ≤ 0.00001) where gains and losses indicate relative increases or decreases in copy number, respectively, in GCT relative to GMT; and panel (C) shows GO terms enriched among genes associated with DMRs (*P* ≤ 0.05), where gains indicate hypermethylated DMRs and losses indicate hypomethylated DMRs in GCT relative to GMT.

### Case study II

Parathyroid tumors causing primary hyperparathyroidism are endocrine tumors mostly classified as adenomas (85%), which affect women more often than men usually when they are beyond their fourth decade of life [72]. The initial manifestation of the disease involves renal stones, reduced bone mineral density, psychiatric symptoms, and hypertension [73]. Parathyroid cancer is frequently associated with persistent signs and symptoms of hypercalcemia [74]. This tumor is considered of high morbidity [75], causing overproduction of parathyroid hormone with very high calcium levels. The pathogenesis of this disease has been associated with genetic variants in several genes [76, 77] and studies have shown methylation alterations driven by parathyroid adenomas [78]. A comprehensive methylomic analysis of malignant parathyroid tumors revealed aberrant methylation in 367 genes [79].

Currently, the only definitive treatment for these tumors involves surgical removal, namely parathyroidectomy [80]. Medicines that decrease parathyroid hormone and calcium levels (i.e., calcimimetics) are a potential alternative either for patients not suitable for parathyroidectomy or in patients with very high parathyroid hormone and/or calcium levels prior to surgery. Little is known about the effects of calcimimetics in the parathyroid glands. However, it is known that the treatment of another neuroendocrine tumor, that is, human pancreatic carcinoids, with the also receptor‐binding drug valproic acid leads is mediated by epigenetic alterations [81].

In this study, genome and epigenome profiles of human parathyroid tissues were compared between patients medicated with the calcimimetic drug “Mimpara” (defined as treatments; PT) and patients not treated with Mimpara (defined as controls; PC). Mimpara works by activating calcium‐sensing receptors in order to inhibit parathyroid hormone secretion [42], and involves a reduction in the production of parathyroid hormone [82]. The objective of this case study was to identify to which extent the effect of Mimpara on parathyroid tumor cells is mediated by epigenetic and/or genetic alterations.

The application of our GBS‐MeDIP method led to the initial identification of 72 111 SNPs among all the analyzed samples from PT (*n* = 8) and PC (*n* = 17) individuals (Fig. 5A). After filtering parameters were applied, 46 663 SNPs remained and were used to perform a PCA analysis (Fig. 5B). None of these SNPs involved fixed alleles between PT and PC individuals. The PCA plot (Fig. 5B) shows that the samples are not randomly distributed among treatments, since three PT samples exhibit clustering. This observation suggests that the genetic background of three individuals is different from the other participants in the study. This information is relevant for the interpretation of the epigenetic analysis, since it is expected that differences in genetic background would also imply epigenetic differences that are not in principle related to the treatment. The majority of the SNPs are in introns, promoters and intergenic regions (Fig. 5C).

**Fig. 5:**
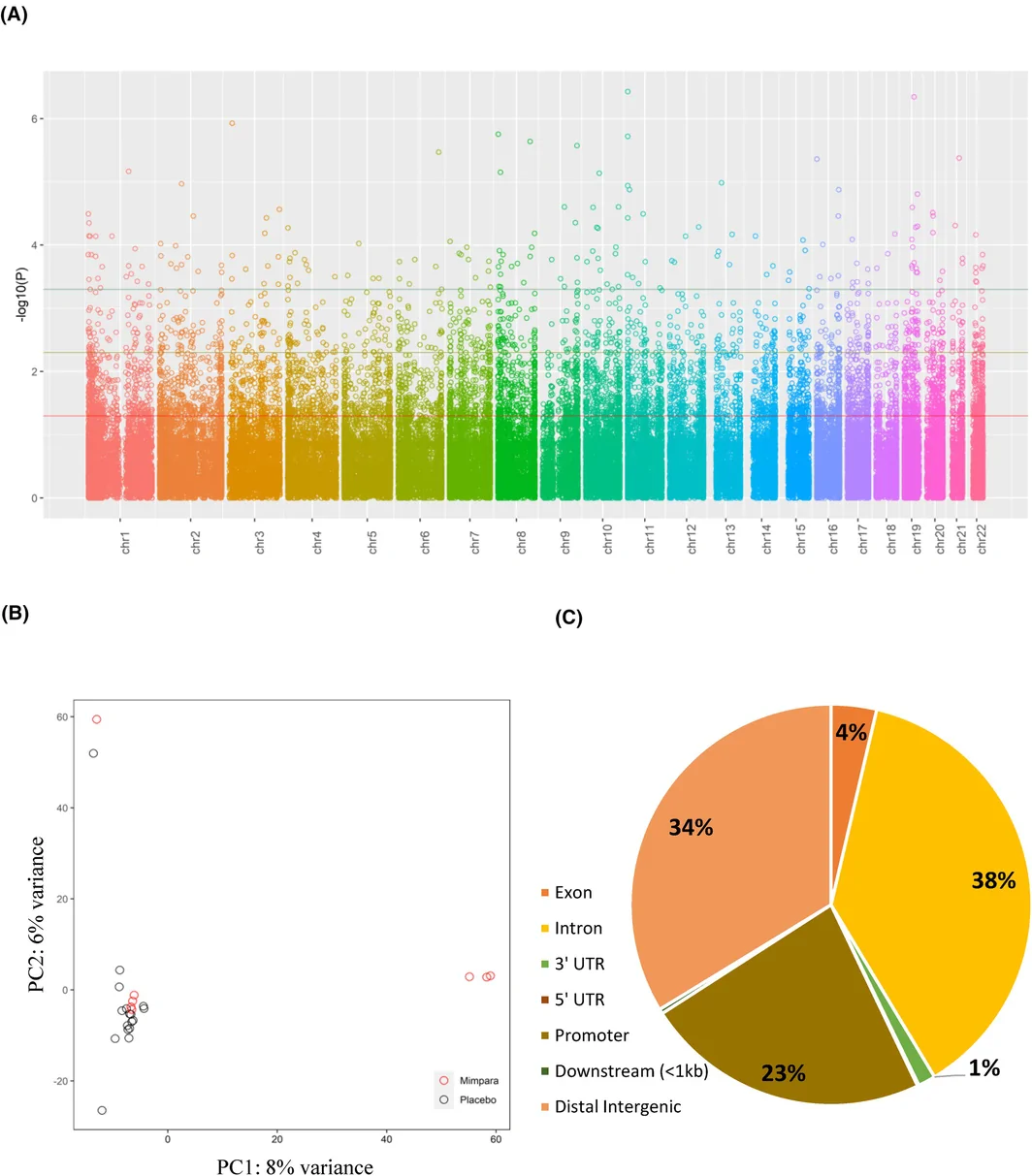
Plots related to the single nucleotide polymorphisms (SNPs) identified between parathyroid tumor (PT) and paired control (PC) DNA samples from 25 individuals. (A) Manhattan plot showing the SNPs identified with different levels of significance (green line indicates a *P*‐value of 0.05 for nominal significance, yellow line for a *P*‐value of 0.005 indicating higher significance, and red line for a *P*‐value of 0.1, suggesting an exploratory threshold); (B) principal component analysis (PCA) based on all the 46 663 SNPs identified; and (C) pie chart showing the functional annotation of the SNPs identified (*P* ≤ 0.05).

Because of the importance of CNVs for the etiology of cancer, CNVs were investigated between the PT and PC DNA samples. A total of 56 494 CNVs (p value, Fig. 6A) were identified, which corresponds to 8% of the genome. Differences in copy number levels across individuals are shown in Fig. 6B for the CNVs with significance *P* ≤ 0.00001 value. Modules of CNVs are clearly visible between PT and PC. The majority of the CNVs are in promoter regions, followed by introns and exons (Fig. 6C), a pattern that differs from that of SNPs.

**Fig. 6:**
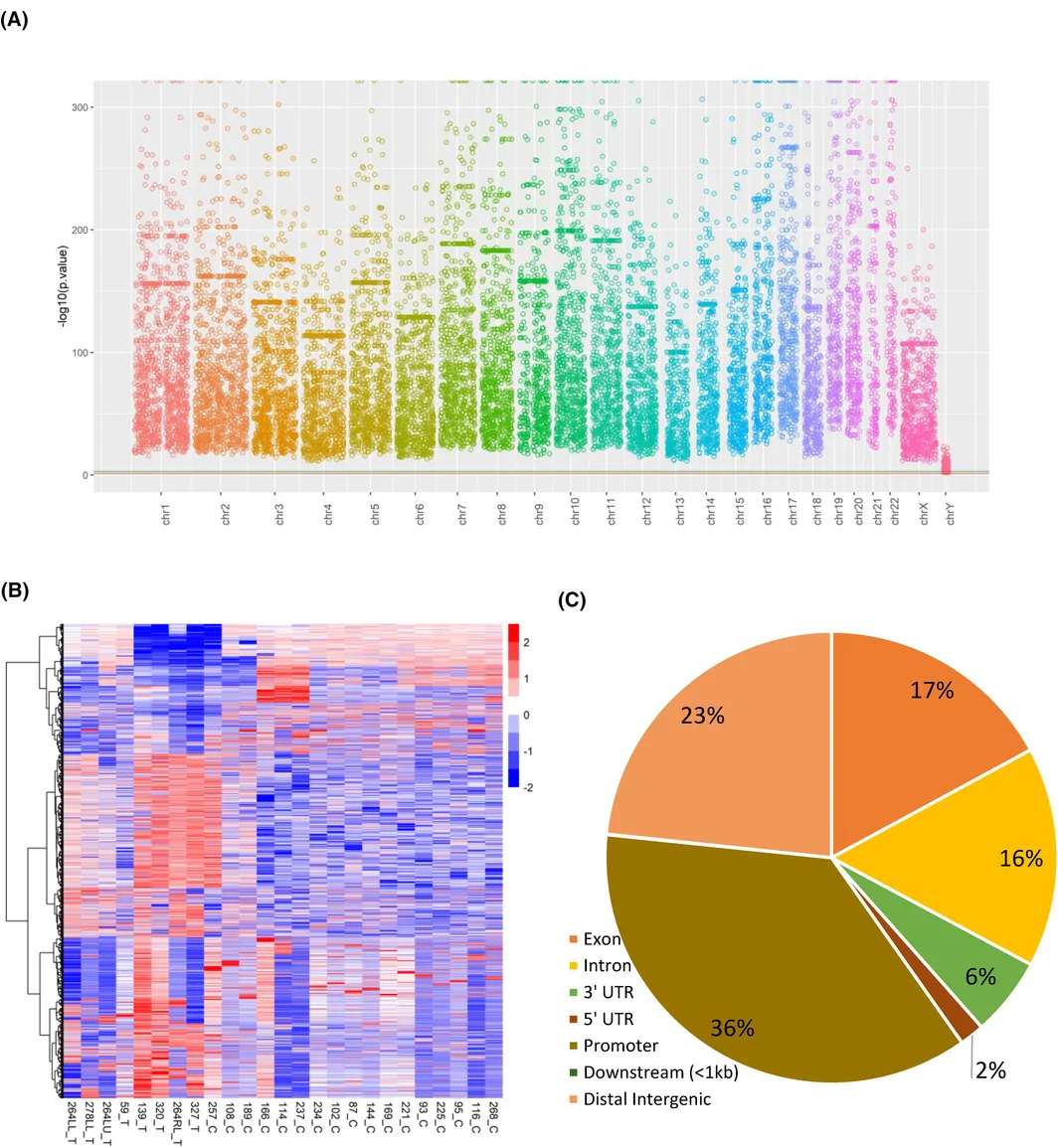
Plots related to the copy number variations (CNVs) identified between parathyroid tumor (PT) and paired control (PC) DNA samples from 25 individuals. (A) Manhattan plot showing the CNVs identified with different levels of significance; (B) Heat map showing the differences in copy number levels across individuals for CNVs obtained with *P*‐value ≤ 0.00001 (the scale to the upper right of the plot represents CNV intensity on a log2 scale, indicating relative copy number changes); and (C) pie chart showing the functional annotation of the same CNVs identified.

Next, a differential methylation analysis between the DNA samples from the PT and PC individuals was performed. A total of 9939 regions of interest (ROIs) were retained employing a ROI‐level FRD threshold of ≤ 0.05. Differential comparison between PT and PC within these ROIs identified 239 DMRs at nominal *P* ≤ 0.05, which 19 remained significant after FDR correction (FDR ≤ 0.05; Table 2). The DMRs related to the different levels of significance are shown in Fig. 7A. Interestingly, all the DMRs identified with *P*‐value ≤ 0.005 were hypermethylated in the PT group (relative to the PC samples) (Fig. 7a). The methylation levels across tissue types are shown in Fig. 7B for the DMRs with significance *P*‐value ≤ 0.05. Most of the DMRs (*P* < 0.05) were located in intronic (50%) followed by distal (30%), promoter (13%), exonic (4%), and 3′UTR (%3) (Fig. 7C). The identified DMRs were evenly distributed across the chromosomes of the human genome (Fig. 7D). In Fig. S2b, hierarchical clustering within the heatmap effectively separates the PC, given placebo, from the PT group, exhibiting distinct methylation patterns between the PT and PC groups. The uniformity in the placebo group contrasts with the diverse epigenetic responses observed between groups, indicating distinct methylation profiles between groups.

**Table 2:** Genomic features and annotations for the identified DMRs (FDR ≤ 0.05) between PT and PC DNA samples from 25 individuals. The table includes location, size, CpG count, methylation changes (logFC), statistical significance (FDR), functional context (annotation), and gene identifiers (SYMBOL and GENE NAME).

Genome location	Width	CpG count	logFC	FDR	Annotation	Symbol	Gene name
chr1:46255307–46 255 624	318	3	4.5	0.040	Intron	RAD54L	RAD54 like
chr11:43955732–43 956 183	452	3	4.2	0.040	Intron	C11orf96	Chromosome 11 open reading frame 96
chr12:32287957–32 288 340	384	1	4.0	0.036	Intron	BICD1	BICD cargo adaptor 1
chr14:20911179–20 911 438	260	5	2.4	0.049	Intron	ECRP	Ribonuclease A family member 2 pseudogene
chr15:75756678–75 757 023	346	7	4.2	0.015	Promoter (1‐2 kb)	LOC101929408	Uncharacterized LOC101929408
chr16:11183631–11 183 868	238	6	4.5	0.005	Distal Intergenic	LOC107984859	Uncharacterized LOC107984859
chr17:5302316–5 302 663	348	8	4.2	0.036	Intron	RABEP1	Rabaptin, RAB GTPase binding effector protein 1
chr19:18951841–18 952 254	414	5	4.5	0.020	Distal Intergenic	HOMER3	Homer scaffold protein 3
chr22:43445636–43 445 951	316	5	4.3	0.031	Intron	MPPED1	Metallophosphoesterase domain containing 1
chr22:45605875–45 606 118	244	4	5.3	0.000	Promoter (<=1 kb)	LINC01589	Long intergenic non‐protein coding RNA 1589
chr3:4869274–4 869 622	349	9	4.5	0.022	Intron	BHLHE40‐AS1	BHLHE40 antisense RNA 1
chr3:51569910–51 570 258	349	6	5.0	0.007	Intron	RAD54L2	RAD54 like 2
chr5:57226635–57 226 879	245	5	3.8	0.029	Intron	GPBP1	GC‐rich promoter binding protein 1
chr5:180857573–180 857 854	282	6	3.9	0.003	Promoter (2‐3 kb)	ZFP62	ZFP62 zinc finger protein
chr6:67662539–67 662 829	291	11	5.5	0.018	Distal Intergenic	LOC105377845	Uncharacterized LOC105377845
chr7:102489568–102 489 907	340	3	4.9	0.029	Intron	RASA4B	RAS p21 protein activator 4B
chr9:83583684–83 584 252	569	10	3.4	0.036	Distal Intergenic	IDNK	IDNK gluconokinase
chr9:136716048–136 716 388	341	9	4.8	0.005	Intron	DIPK1B	Divergent protein kinase domain 1B
chrX:85529801–85 529 997	197	2	2.4	0.036	Distal Intergenic	POF1B	POF1B Actin binding protein

**Fig. 7:**
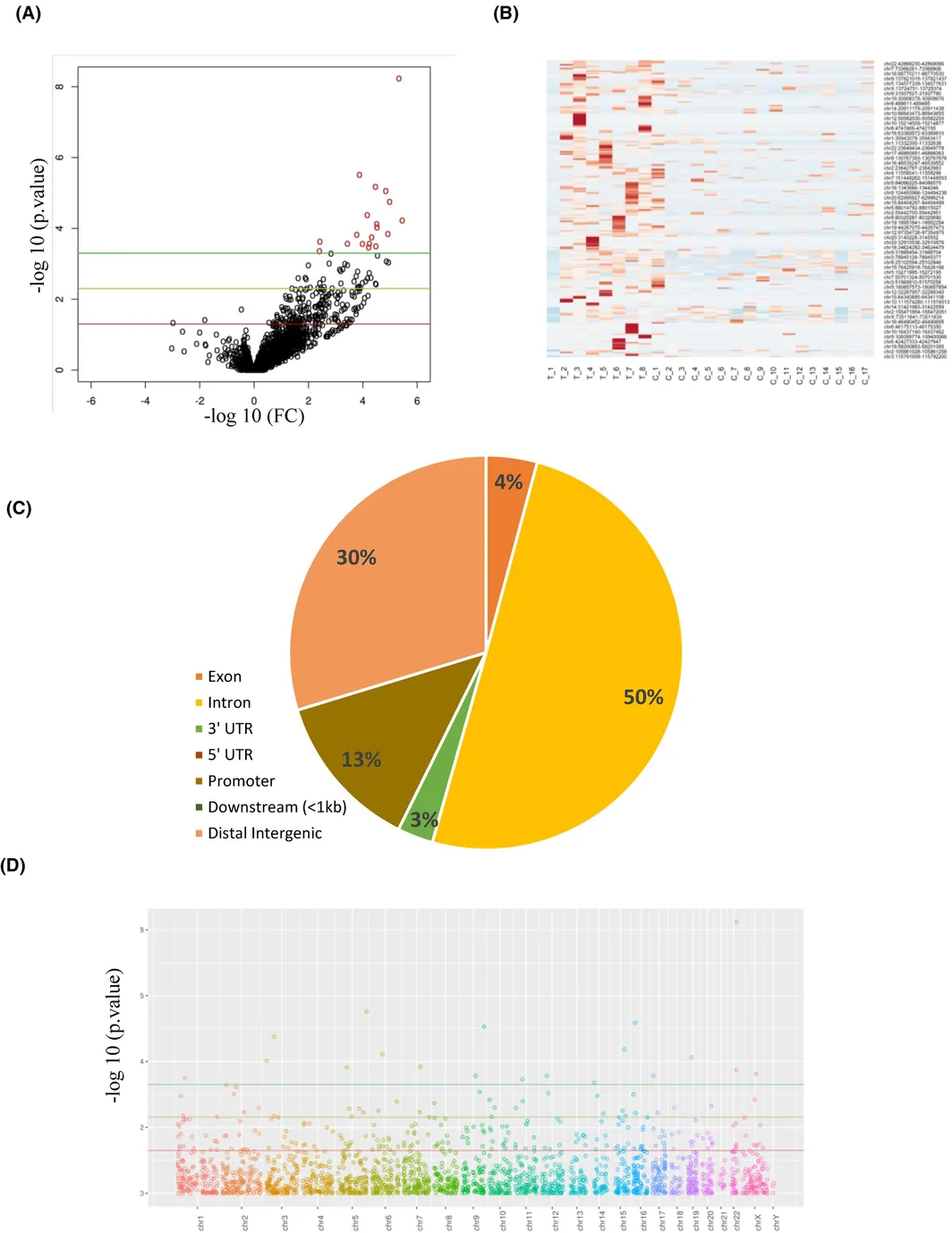
(A) Volcano plot depicting the differentially methylated regions (DMRs) identified at different levels of significance between parathyroid tumor (PT) and paired control (PC) DNA samples from 25 individuals. Positive logFC indicates that PT are hypermethylated in relation to PC; thresholds correspond to *P*‐values 0.05 (red), 0.005 (yellow), and 0.0005 (green). (B) Heat map depicting methylation changes in significant DMRs (*P* ≤ 0.05); the heatmap colors show relative methylation: red for areas of hypermethylation, blue for hypomethylation, and white indicating average methylation levels across samples, reflecting the normalized baseline. (C) Pie chart representing the functional annotation of the identified DMRs (*P* ≤ 0.05). (D) Manhattan plot showing the distribution of DMRs across the chromosomes of the human genome.

To investigate the relationship among the genetic and epigenetic features identified in the parathyroid dataset, genomic overlaps among significant SNPs, CNVs, and DMRs were examined. Of 4112 SNPs, 124 (3.02%) were located within CNV regions and seven (0.17%) overlapped a DMR. Among the 239 DMRs, 18 (7.53%) overlapped CNVs (Table S2), while no SNP simultaneously overlapped both a CNV and a DMR. At the gene‐annotation level, 2889, 9072, and 236 genes were associated with SNPs, CNVs, and DMRs, respectively. Among these, 1214 genes were shared between SNPs and CNVs, 54 between SNPs and DMRs, and 128 between CNVs and DMRs, while 33 genes were represented across all three feature types (Data S2).

Finally, GO pathway analyses were performed for the SNPs identified by the TREND and ALLELIC association tests (*P* ≤ 0.05, Fig. 8A), CNVs (*P* ≤ 0.00001, Fig. 8B) and DMRs (*P* ≤ 0.05, Fig. 8C). Gains represent increases in copy number (CNVs) and in methylation (hypermethylated DMRs) in PT compared to PC, while losses represent reduced copy numbers and hypomethylated DMRs. These analyses revealed distinct biological pathways associated with each type of genetic or epigenetic variation. Functional convergence was also investigated by identifying overlapping genes associated with the CNVs, SNPs, and DMRs. GO Biological Process enrichment analysis followed by FDR correction identified significant functional enrichment among these overlapping genes, comprising 23 significantly enriched GO terms (Data S2; Fig. S3). Semantic reduction of redundant GO terms grouped these into seven representative functional clusters (Data S2; Fig. S5a). Functional convergence was also investigated in overlapping genes in only two of the datasets, resulting in a set of 1330 multi‐feature‐supported genes. This analysis identified 35 significantly enriched GO terms, which were grouped into nine representative functional clusters, including processes related to glutamate receptor signaling, learning, cell morphogenesis, cell junction assembly, sensory system development, synaptic transmission, and neurotransmitter receptor activity (Data S2; Fig. S5b).

**Fig. 8:**
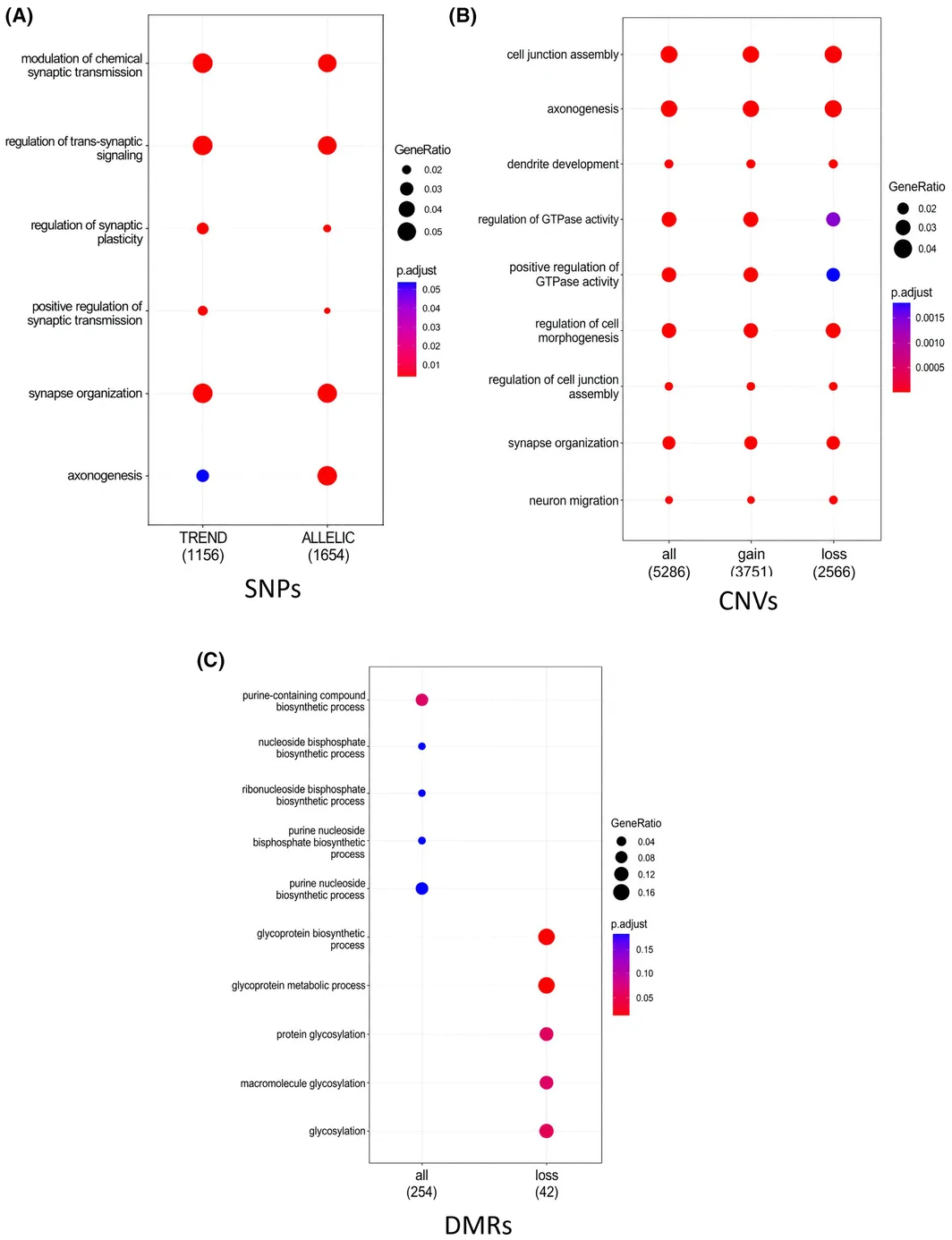
Gene Ontology (GO) pathway analyses for genomic variants in single nucleotide polymorphism (SNP), copy number variation (CNV), and differentially methylated region (DMR) categories identified between parathyroid tumor (PT) and paired control (PC) DNA samples from 25 individuals. Panel (A) shows GO terms enriched among genes associated with SNPs identified using the TREND and ALLELIC tests with a *P*‐value threshold of ≤ 0.05; panel (B) shows GO terms enriched among genes associated with CNVs (*P* ≤ 0.00001) where gains and losses indicate relative increases or decreases in copy number, respectively, in PT relative to PC; and panel (C) shows GO terms enriched among genes associated with DMRs (*P* ≤ 0.05), where gains and indicate hypermethylated DMRs and losses hypomethylated DMRs in PT relative to PC.

## Discussion

The GBS‐MeDIP enables the simultaneous identification of genetic (SNPs and CNVs) and epigenetic (DNA methylation) variation within the same reduced genome fraction across multiple biological samples. This ability of the GBS‐MeDIP combination makes this method useful to employ in experimental setups where epigenetic and genetic marks associated with particular phenotypes or environmental exposures need to be identified. Importantly, the concurrent identification of these complementary molecular features enables their interpretation within the same biological context, facilitating the investigation of the interplay between genetic and epigenetic mechanisms while identifying genomic regions where underlying genetic variation may influence the interpretation of differential DNA methylation. Because of its cost‐effectiveness, the method can be used for identifying methylomic marks in large populations of individuals. In our initial experiments the GBS‐MeDIP was employed to identify epigenetic marks of environmental exposures, such as in red blood cells of chickens (unlike mammals, these are nucleated in birds) subjected to different rearing conditions [83] or exposure to social isolation stress [52]. Using pigs as an experimental model, epigenetic markers for fertility in sperm have been identified, which are moreover related to the season of the semen collection [59] and epigenetic changes in brain regions of piglets in relation to the environment experienced by the pregnant sows [84]. In relation to the interplay between epigenetic and genetic changes, we employed this method in chickens to correlate genetic variant rates with CpG methylation status in their domestication process [57]. In humans, this method opens several possibilities for the identification of epigenetic marks in large cohorts in relation to environmental exposures, disease susceptibility, or resistance to treatments such as in cancer.

### Comparison of GBS‐MeDIP with other methods

There are fundamentally three major types of Next Generation Sequencing (NGS) methods, which make use of REs in order to digest the genome of target species [85]. These three reduced representation techniques are: GBS, sequencing of reduced representation libraries (RRLs), and restriction site‐associated DNA sequencing (RAD‐Seq) [85]. In relation to the DNA input, GBS has been shown to be the most efficient among these methods, since it can work with very small starting amounts of DNA, such as 100 ng per sample. The latter two sequencing methods require higher amounts of genomic DNA, usually in the microgram range [32, 85]. Moreover, in GBS, this quantity can be further reduced according to the desired number of individuals. In the case of the present protocol, it is important to obtain a minimally recommended amount of pooled DNA to be used as the input (> 500 ng)^25^ for the subsequent MeDIP, which is applied to the pool composed of individual barcoded DNA samples. Although RAD‐Seq enables a better selection of the desired fragment sizes, this technique is slightly more labor‐intensive and costly compared to GBS [33]. RRL, in turn, has the major drawback that it cannot differentiate between samples, as it doesn't employ a barcode system [85]. GBS is a simple and robust technique developed by Elshire et al. in maize [32], which can be used for breeding, population studies, or even trait mapping to define genetic variants in many species [47, 86]. Another benefit of GBS is the reduced sample handling steps required, since the digestion and ligation steps are performed in the same well of a PCR plate and the DNA purification steps are performed in the pools and not in individual samples [32]. Moreover, the fact that GBS does not have an extra fragment selection step, such as in RAD‐Seq, is compensated by the subsequent step of our protocol, in which only fragments selected by methylated DNA immunoprecipitation will be part of the final library.

Different high‐throughput techniques have been developed for genome‐wide DNA methylation profiling of a reduced number of CpGs in the genome. These include methylated DNA immunoprecipitation (MeDIP), reduced representation bisulfite sequencing (RRBS) [30], and Illumina Infinium chip beads [87]. Each of these methods has its own strengths and weaknesses [88].

RRBS, which is widely employed for genome‐wide methylation analysis, relies on the use of methylation sensitive/insensitive restriction enzymes (REs) that differentially cut the CpG site on the 5´CCGG 3′ motif depending on its methylation state (i.e., *HpaII* and *MspI*) [89]. Because 94% of the CpG islands in the human genome contain this motif, RRBS DNA methylation analysis are biased towards CpG islands [89]. Despite having different CpG coverage, resolution, quantitative accuracy, efficiency and cost, these methods produce comparable methylation calls [90].

In the development of the GBS‐MeDIP, the GBS was chosen due to its practicality, ability to interrogate multiple samples at the same time, and low DNA input required, while the MeDIP because of its ability to capture genomic fragments for the detection of methylated regions across the entire genome of different species [91]. The combination of these two techniques proved to be uniquely complementary for many reasons: (i) the RE employed in the protocol creates digested DNA fragments with overhangs of known sequence, which then allows a multiplex approach to generate both the genomic and methylomic libraries; (ii) while GBS enriches for fragments shorter than the desired length size for sequencing, MeDIP (performed on the GBS libraries composed of pooled individual DNA samples) eliminates these fragments during the antibody immunoprecipitation step; (iii) the enrichment of the appropriate DNA fragment size is progressive in the protocol, with both the RE and MeDIP (in addition to the PCR and purification steps) contributing to create a library of a desired range of fragment sizes; (iv) both procedures also contribute to the enrichment of methylated regions since the digestion with *PstI* already enriches for CpG‐rich regions [29], providing a preselection for the later action of the antibody against methylated cytosines. Thus, the GBS‐MeDIP presented here is a simple and cost‐efficient method for the interrogation of a reduced genomic fraction and its corresponding methylome. While the PstI‐based digestion used in GBS naturally yields fragments located in relatively CpG‐rich genomic contexts, the method does not specifically target CpG islands. The distribution patterns observed here confirm that the genomic fraction captured by our approach is enriched in genic regions (intronic, promoter, and exonic), reflecting the genomic locations where CpGs and methylation are most concentrated.

### Limitations of the GBS‐MeDIP

In general, there are two main limitations of our method. One is the small number of CpGs interrogated relative to those present in the whole genome (reduced representation), and second, that it does not allow us to analyze the methylation status of individual CpGs within the same sequenced fragment. This is due to the fact that the representation of DNA methylation is based on fragments produced by PstI restriction and then immunoprecipitated (by the anti‐methyl cytosine antibody) rather than on specific CpGs, unless a read contains only one CpG. However, the objective of our method is not to identify differentially methylated individual CpGs, but rather regions of the DNA that will subsequently be validated by methodologies that investigate CpG methylation on a local level (e.g., common bisulfite sequencing).

### Conclusions from the case studies

In the present paper, the GBS‐MeDIP method has been employed in two different sets of samples: in human biopsies of (i) patients with gastric cancer to compare cancerous vs marginal samples, and (ii) parathyroid tumors from patients treated or untreated with Mimpara. The objective was to apply the GBS‐MeDIP method for the simultaneous identification of epigenetic and genetic biomarkers in oncology research.

In both case studies, the GBS‐MeDIP succeeded in identifying a number of epigenetic (DMRs) and genetic changes (SNPs and CNVs) between experimental groups, with variable statistical significance. This identification of epi/genomic markers is relevant both to provide a set of genomic regions where more in‐depth analyses can be performed individually, and/or may be used for downstream analyses to shed light on the pathways affected in each scenario (e.g., GO pathway analyses).

A similar number of SNPs obtained from the GBS‐MeDIP data were identified in both case studies. In case study I, a PCA based on 71 061 SNPs revealed that some tumoral samples have already differentiated in genetic composition compared to the marginal cells. This might mean that these tumors could be in a more advanced progression stage than the others. In case study II, a PCA on 72 111 SNP data revealed that the genetic background differed among the study individuals. This information is important to identify outliers in the study population, and genetic composition associated with specific phenotypes, as well as to provide a way to control whether epigenetic differences between experimental groups may or may not be derived from genetic differences. The genomic functional effects of the SNPs are very similar in both case studies, with SNPs present mostly in introns, followed by intergenic and promoter regions.

Unlike the SNPs, CNVs found in case study II nearly double those found in case study I. This information could point towards a possible relation of the Mimpara treatment with copy number variations. However, it could also reflect a different genetic background of the tumors since these patients generally had higher parathyroid hormone levels requiring preoperative treatment with Mimpara. Although the genomic functional effects of CNVs are very similar in both case studies, they differ considerably from those of SNPs, mainly in the reduced presence of CNVs in introns and their increased presence in promoter regions compared to SNPs.

In relation to DMRs, many more passed FDR in case study II than in case study I. Moreover, a tendency towards hypermethylation in PT samples is prominent, with all the DMRs that passed FDR being hypermethylated in PT samples. In case study I, only four DMRs passed FDR, being two hyper and two hypomethylated in GCT samples compared to GMT samples. It is interesting to note that the functional annotation of DMRs differs substantially between both case studies.

Generally, we found that each genomic level investigated (i.e., CNVs, SNPs, or DMRs) associated with a particular set of pathways. However, functional convergence was identified in a subset of overlapping genes associated with the markers identified across omic levels, although the pattern differed between the two case studies. In the gastric dataset, functional convergence was observed, with the identified SNPs, CNVs, and DMRs mapping to overlapping genes involved in processes such as mitochondrial transport and apoptotic signaling, mRNA stability, synapse maturation, and Golgi organization. However, when the analysis included genes shared among two datasets, rather than across all three, no GO terms remained significantly enriched after FDR correction. In the parathyroid dataset, functional convergence was observed only in genes overlapping between SNPs and CNVs, highlighting processes associated with neuronal signaling and development, cell adhesion and junction organization, and regulation of transmembrane signaling. Together, these findings illustrate how the complementary molecular features identified by GBS‐MeDIP can converge at the level of biological function even when direct genomic overlap between feature types is limited. Importantly, such functional convergence indicates biological co‐occurrence rather than dependency or causal relationships between genetic and epigenetic variation.

Both case studies show CNVs overlapping with DMRs. Thus, this combination of analysis allows for the identification of confounding genetic factors when investigating epigenetic effects. In the present case, the DMRs overlapping with CNVs could be singled out as potential confounding regions. These observations would not be possible if the genomic and methylomic levels are not simultaneously investigated.

In addition to providing tools for controlling artifacts related to epigenetic effects, genetic information can be used to identify specific SNPs or CNVs in relation to phenotypes of interest, provided that a sufficient number of individuals is used to ensure appropriate power. In this sense, the cost‐effectiveness of GBS‐MeDIP allows to expand studies to numbers of individuals needed for genome‐wide association analyses with sufficient power. The simultaneous identification of DMRs, SNPs, and CNVs in multifactorial diseases like cancers opens a promising alternative to better understand their molecular etiology.

## Conflict of interest

The authors declare no conflicts of interest.

## Author contributions

Conceptualization: FP and CG‐B; Methodology and sample collection: FP, SR, JU, OG, TK; CG‐B; Biological Resources: OG, MAHF; Data analysis: FP; Writing of original draft: SR, FP and CG‐B Review & editing: FP, CG‐B, LLC, JU, OG, TK; MAHF and SR Final version: CG‐B Funding acquisition: CG‐B, LLC, and MAHF.

## Supporting information


**Fig. S1.** *In silico* digestion prediction of six different reference genomes using the PstI RE.
**Fig. S2.** Hierarchical clustering heatmaps displaying individual‐to‐individual comparison of methylation profiles.
**Fig. S3.** GO Biological Process enrichment among genes shared across genetic and epigenetic feature classes.
**Fig. S4.** Functional convergence in the gastric dataset. Network representation of 70 significantly enriched GO Biological Process terms (FDR ≤ 0.05) among genes shared across SNPs, CNVs, and DMRs.
**Fig. S5.** Functional convergence among genetic and epigenetic features in the parathyroid dataset.
**Table S1.** Descriptive statistics for significant DMRs (adj. *P* value < 0.5) overlapping with CNVs (with *P* value ≤ 0.00001) found in gastric biopsies.
**Table S2.** Descriptive statistics for significant DMRs (adj. *P* value < 0.5) overlapping with CNVs (with *P* value ≤ 0.00001) in parathyroid samples.

**Data S1.** Genomic distribution and CpG characteristics of DMRs and CNVs identified using GBS‐MeDIP. Comparative analysis of gastric and parathyroid tumor datasets, including the number and proportion of differentially methylated regions (DMRs) and copy number variations (CNVs) across genomic annotation categories, differences in their genomic distributions, and CpG content and density across genomic features.

**Data S2.** Genomic feature overlaps and functional enrichment analyses of SNPs, CNVs, and DMRs in human tumor datasets. Summary of genetic and epigenetic feature counts, overlapping genes among single nucleotide polymorphisms (SNPs), copy number variations (CNVs), and differentially methylated regions (DMRs), and Gene Ontology (GO) enrichment analyses, including semantic clustering and representative functional terms in gastric and parathyroid tumor datasets.

## Data Availability

All data and computational resources utilized in our study are fully accessible. The dataset underpinning our findings is deposited in the European Nucleotide Archive (ENA) repository, EMBL‐EBI, with accession number PRJEB35669, available at ENA repository link (www.ebi.ac.uk/ena/data/view/PRJEB35669). Comprehensive details on the computational methods, including all programs, R packages, and scripts, are provided through our methodological paper and are openly accessible [28]. For a detailed exposition of our GBS‐MeDIP protocol development, along with the accompanying pipeline and bioinformatics approach, please refer to our GitHub repository at GBSMeDIP GitHub repository (https://github.com/fpertille/GBSMeDIP.git).
