# Supplementary material for: GBS‐MeDIP: A method for the simultaneous identification of genetic and epigenetic variants in tumors
Source: FEBS Open Bio. 2026 Nov 9:10.1002/2211-5463.70360. Online ahead of print. doi: 10.1002/2211-5463.70360 (PMC13749004; doi:10.1002/2211-5463.70360)
Supplement: Supplementary file 1 [file FEB4-9999-0-s001.pdf]

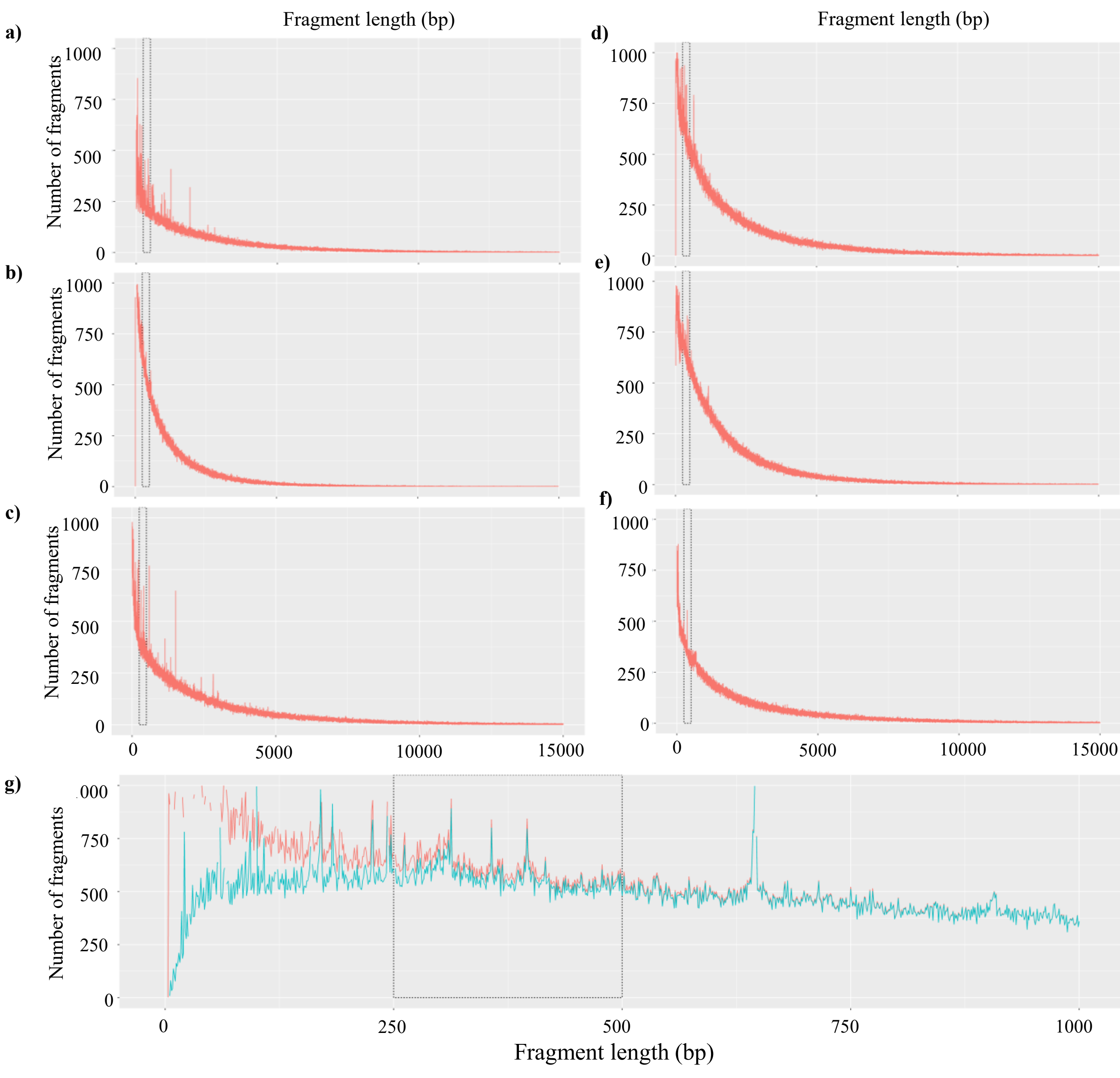

Supplementary Figure S1: In silico digestion prediction of six different reference genomes using the PstI RE. Fragment lengths are represented in red for: (a) D.rerio; (b) G. gallus; (c) M. musculus; (d) H. sapiens; (e) S. scrofa and (f) C. familiaris reference genomes. (g) shows the range of 250-500 (black dotted box bp) after in silico digestion of the H. sapiens reference genome (blue) considering fragments containing at least one CpG in their composition.

a)

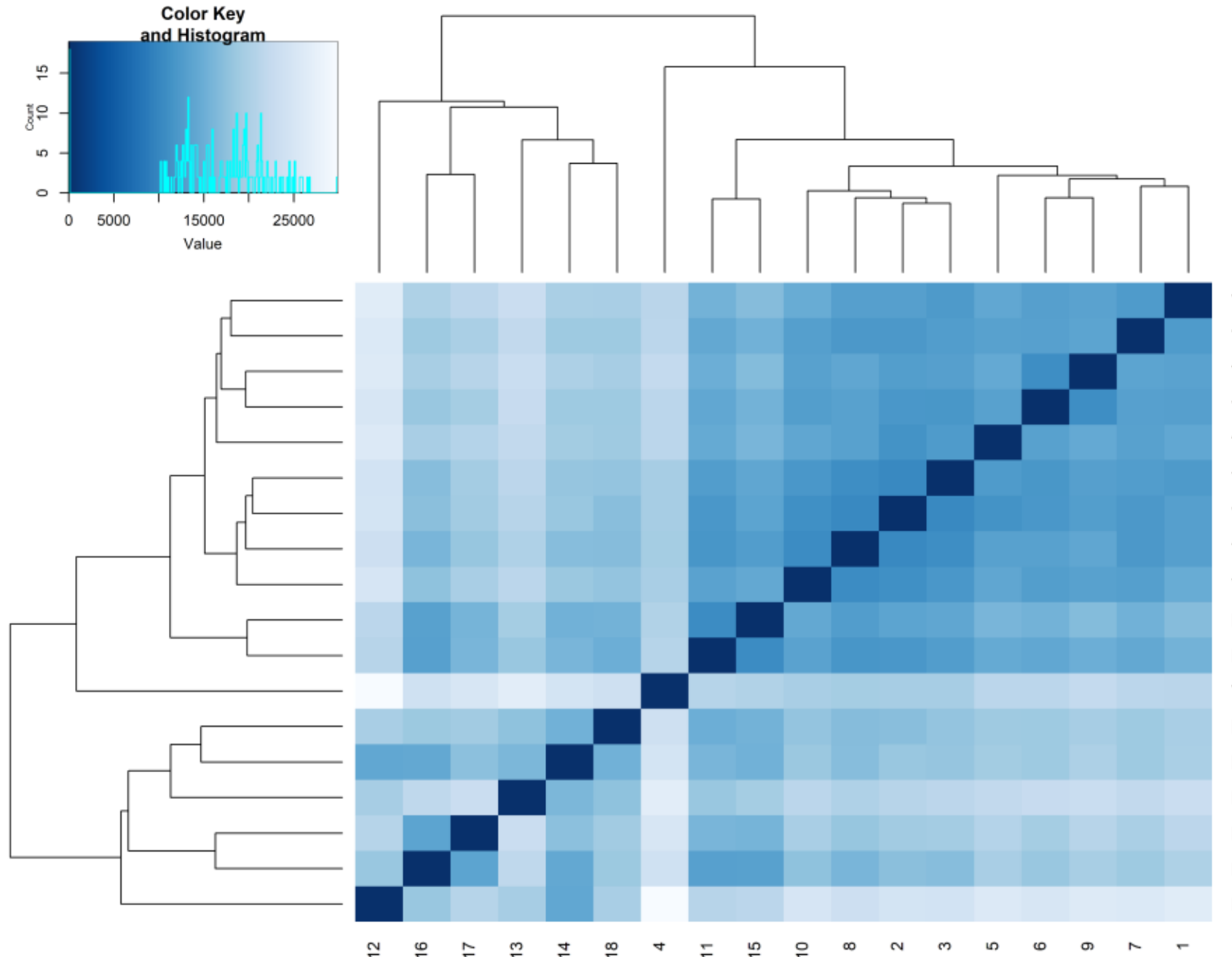

b)

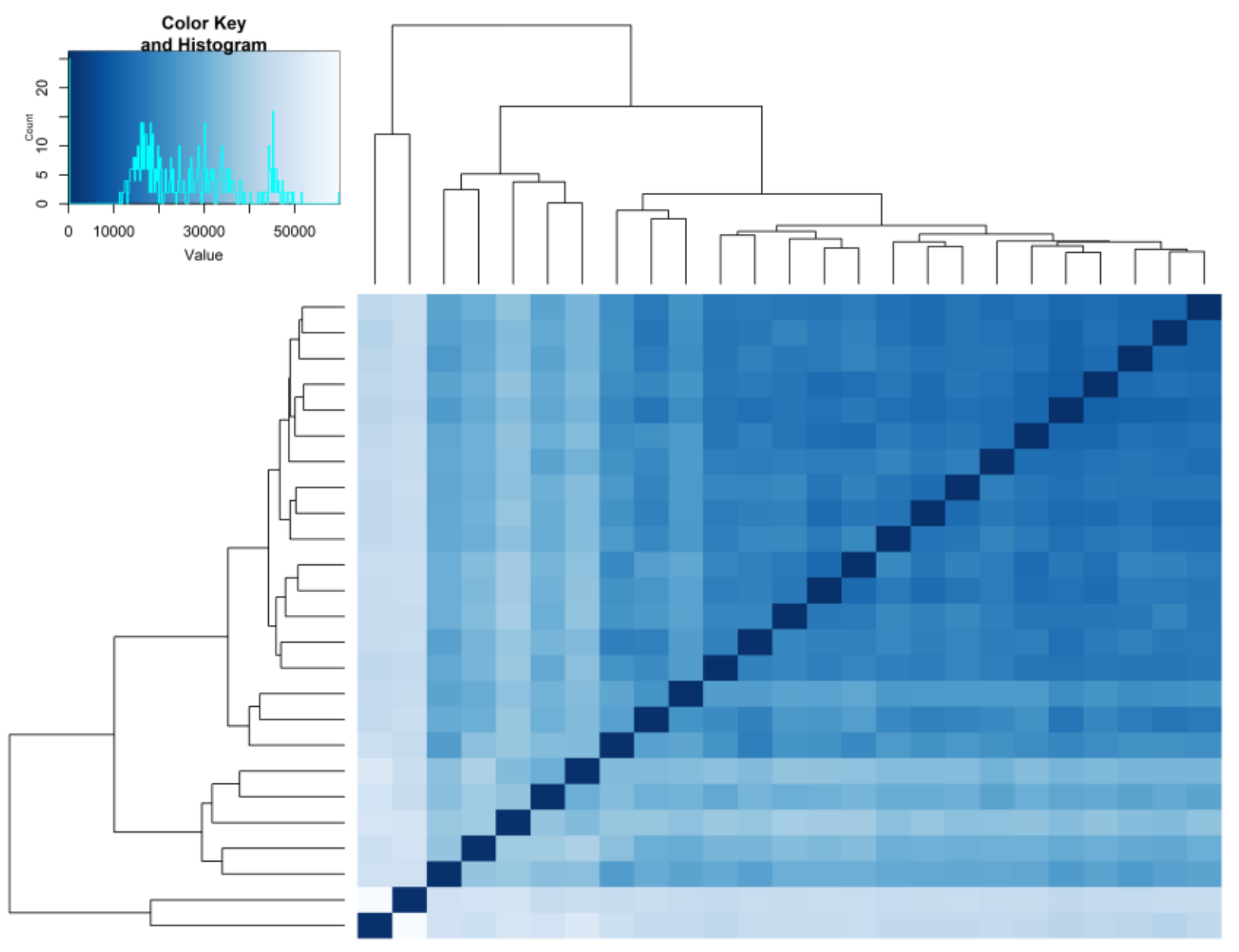

Supplementary Figure S2: Hierarchical clustering heatmaps displaying individual-to-individual comparison of methylation profiles. (a) Heatmap shows the clustering of individual samples into tumor (T) and normal (M) cells, based on differential methylation patterns. (b) Heatmap differentiates individual samples within placebo-controlled (PC) and treated (PT) groups, reflecting methylation status.

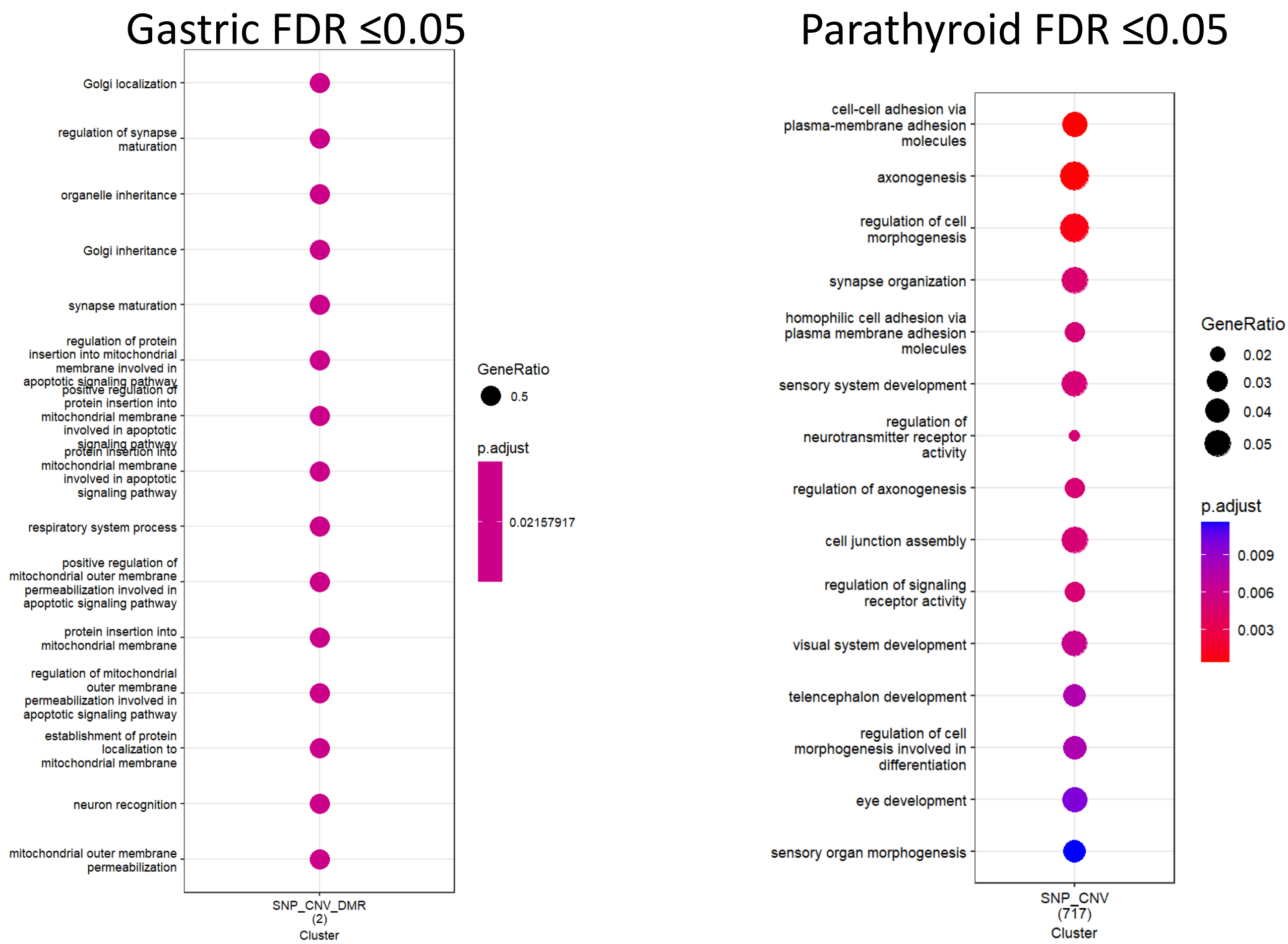

Supplementary Figure S3. GO Biological Process enrichment among genes shared across genetic and epigenetic feature classes. Dot plots show significantly enriched GO Biological Process terms (FDR ≤ 0.05) for shared gene sets in the gastric and parathyroid datasets. Dot size represents the gene ratio and color indicates the adjusted P-value. Significant enrichment was identified for the SNP–CNV–DMR shared gene set in the gastric dataset and the SNP–CNV shared gene set in the parathyroid dataset.

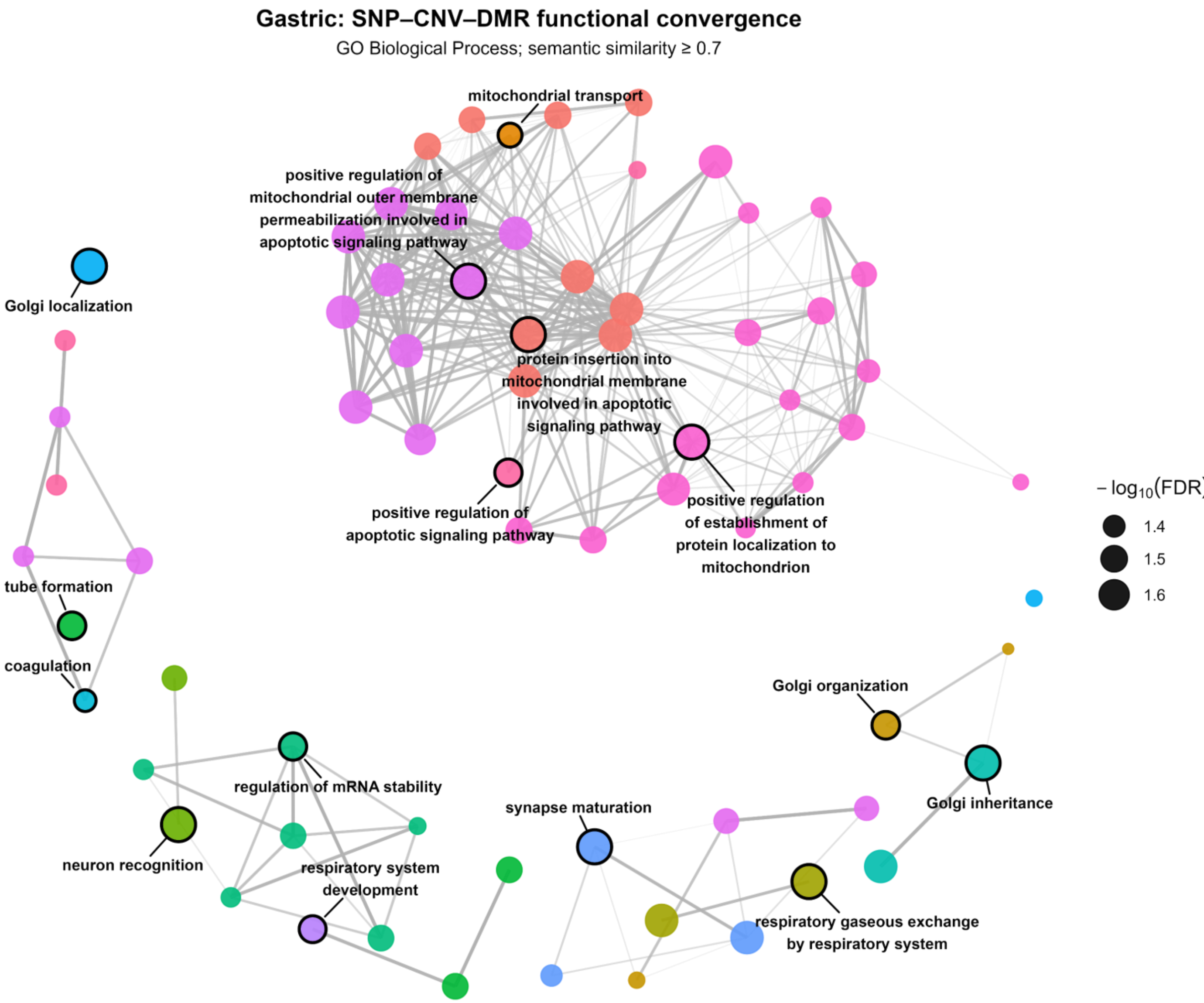

Supplementary Figure S4. Functional convergence in the gastric dataset. Network representation of 70 significantly enriched GO Biological Process terms (FDR ≤ 0.05) among genes shared across SNPs, CNVs, and DMRs. Nodes represent GO terms, edges indicate semantic similarity ≥ 0.7, and colors indicate semantic clusters. Black-outlined nodes indicate the 15 representative terms.

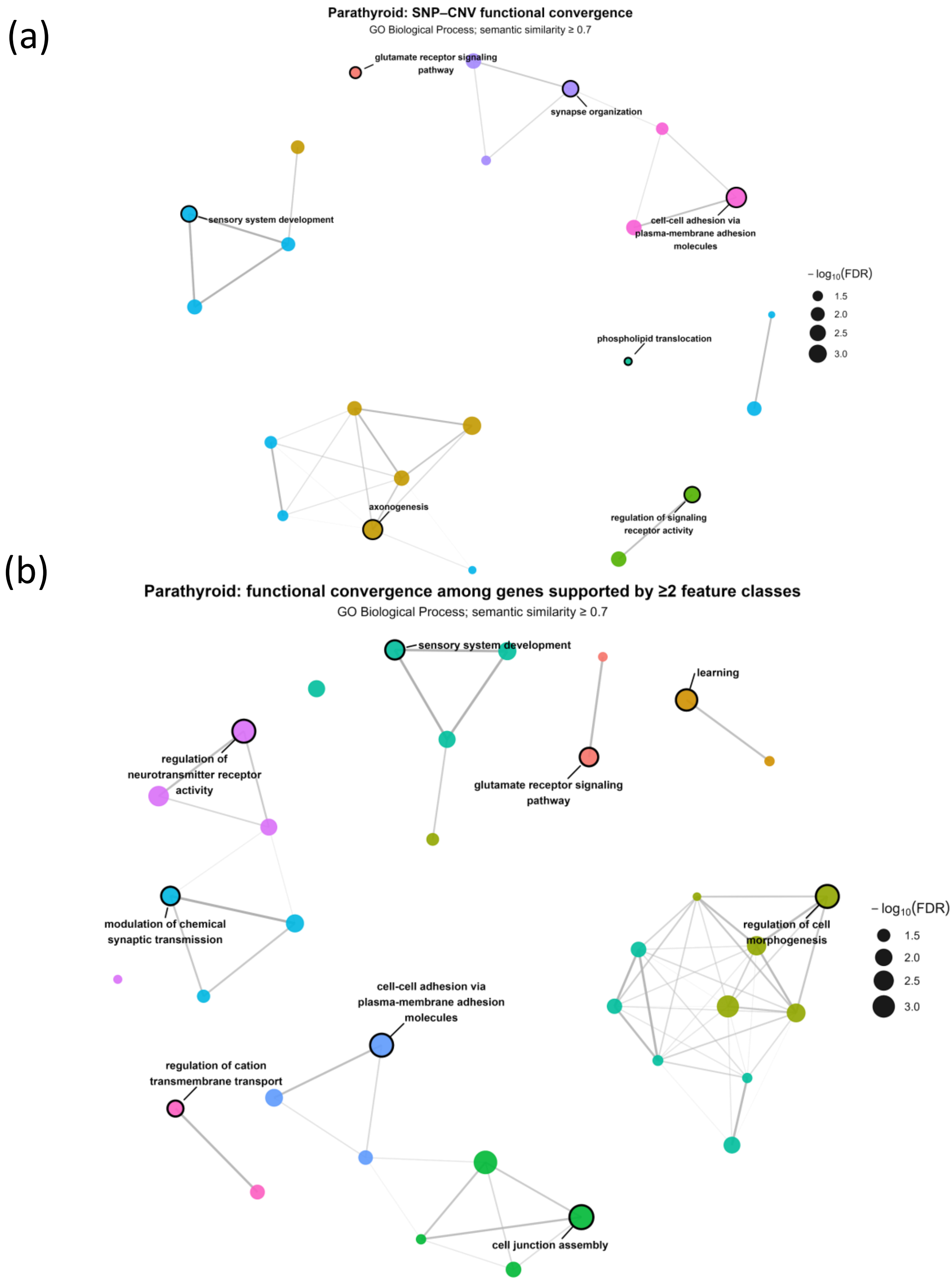

Supplementary Figure S5. Functional convergence among genetic and epigenetic features in the parathyroid dataset. (a) Network representation of 23 significantly enriched GO Biological Process terms (FDR ≤ 0.05) identified among genes shared between SNPs and CNVs, grouped by semantic similarity into seven representative functional clusters. (b) Broader cross-feature analysis including 1,330 genes represented in at least two feature classes (SNP, CNV, and/or DMR), identifying 35 significantly enriched GO Biological Process terms grouped into nine representative functional clusters. In both networks, nodes represent significantly enriched GO terms, node size corresponds to −log10(FDR), and edges connect terms with Rel semantic similarity ≥ 0.7. Node colors indicate semantic clusters, and black-outlined nodes indicate the representative GO term selected for each cluster based on the highest −log10(FDR).

Suppl Table S1: Descriptive statistics for significant DMRs (adj. p value < 0.5) overlapping with CNVs (with p-value ≤ 0.00001) found in gastric biopsies

CNV		DMR				
**Genome coordinate**	**State**	**Genome coordinate**	**logFC**	**p.value**	**adj. p. value**	**CG content**
Chr9-41653790-41675044	GAIN	chr1:23829499-23829897	3.24	1.68E-02	1.00E+00	6
chr4-134165216-134191784	GAIN	chr12:7251722-7251979	3.26	2.44E-02	1.00E+00	6
chr18-12479930-12517126	GAIN	chr18:12504975-12505164	1.86	4.97E-02	1.00E+00	3
chr12-7235012-7256266	GAIN	chr19:53963691-53963850	2.56	8.55E-03	1.00E+00	3
chr22-33778442-33799696	GAIN	chr19:57751722-57751983	4.83	1.49E-02	1.00E+00	3
chr6-156117350-156138604	LOSS	chr22:33796648-33796896	4.55	2.13E-02	1.00E+00	4
chr8-97041612-97078808	GAIN	chr4:134176946-134177349	4.24	1.95E-02	1.00E+00	3
chr1-23814692-23835946	LOSS	chr6:156129331-156129592	4.21	2.00E-02	1.00E+00	0
chr19-53961014-54014152	GAIN	chr8:97044786-97045105	2.64	3.00E-04	2.71E-01	5
chr19-57733954-57755208	GAIN	chr9:41654462-41654603	0.57	4.47E-02	1.00E+00	8

Suppl Table S2. Descriptive statistics for significant DMRs (adj. p value < 0.5) overlapping with CNVs (with p-value ≤ 0.00001) in parathyroid samples

CNV		DMR				
**Genome coordinate**	**State**	**Genome coordinate**	**logFC**	**p.value**	**adj. p. value**	**CG content**
chr2-25683655-25698711	LOSS	chr2:25688418-25688810	3.84	1.75E-02	2.92E-01	15
chr4-16544663-16559719	GAIN	chr4:16557673-16557925	0.86	3.25E-02	3.97E-01	8
chr4-189368723-189383779	GAIN	chr4:189379314-189379619	2.43	1.33E-02	2.66E-01	2
chr5-160099859-160114915	LOSS	chr5:160112096-160112341	3.99	3.51E-03	1.66E-01	4
chr7-159222847-159241667	GAIN	chr7:23891175-23891481	2.68	1.43E-02	2.77E-01	3
chr7-23884463-23907047	LOSS	chr7:159228863-159229066	2.87	4.91E-03	1.83E-01	4
chr8-50680379-50695435	GAIN	chr8:50692552-50692804	0.84	2.80E-02	3.74E-01	5
chr9-25092707-25107763	GAIN	chr9:25102594-25102846	1.28	1.02E-02	2.26E-01	7
chr10-21765331-21784151	GAIN	chr10:21770172-21770506	3.75	2.66E-02	3.69E-01	4
chr11-117220371-117239191	GAIN	chr11:117225914-117226217	2.62	2.00E-02	3.05E-01	4
chr12-50567459-50582515	GAIN	chr12:50582030-50582255	3.34	4.60E-02	4.25E-01	4
chr12-97350215-97369035	GAIN	chr12:97354726-97354975	1.53	5.41E-03	1.86E-01	4
chr13-68167923-68182979	GAIN	chr13:68171173-68171698	1.7	2.19E-02	3.22E-01	14
chr19-30896795-30945727	GAIN	chr19:30908378-30908676	2.98	2.69E-02	3.71E-01	2
chr20-33828951-33859063	LOSS	chr20:33845475-33845739	1.65	4.34E-02	4.24E-01	5
chr22-38823779-38838835	GAIN	chr22:38833337-38833772	3.16	7.60E-03	2.12E-01	13
chr22-42858787-38838835	GAIN	chr22:39859228-39859498	1.55	3.81E-02	3.97E-01	3
chr22-39847587-39862643	LOSS	chr22:42868230-42869066	1.91	3.32E-02	3.97E-01	24
chr2-25683655-25698711	LOSS	chr2:25688418-25688810	3.84	0.017515	0.291622	15
